# Unraveling pathogenesis, biomarkers and potential therapeutic agents for endometriosis associated with disulfidptosis based on bioinformatics analysis, machine learning and experiment validation

**DOI:** 10.1186/s13036-024-00437-0

**Published:** 2024-07-26

**Authors:** Xiaoxuan Zhao, Yang Zhao, Yuanyuan Zhang, Qingnan Fan, Huanxiao Ke, Xiaowei Chen, Linxi Jin, Hongying Tang, Yuepeng Jiang, Jing Ma

**Affiliations:** 1 Department of Traditional Chinese Medicine (TCM) Gynecology, Hangzhou TCM Hospital Affiliated to Zhejiang Chinese Medical University, Hangzhou, China; 2 Research Institute of Women’s Reproductive Health, Zhejiang Chinese Medical University, Hangzhou, China; 3 The Affiliated Hospital of Nanjing University of Chinese Medicine, Nanjing, China; 4 The Third Clinical Medical College, Zhejiang Chinese Medical University, Hangzhou, China; 5 College of Pharmaceutical Science, Zhejiang Chinese Medical University, Hangzhou, China

**Keywords:** Endometriosis, Disulfidptosis, Immunity, Machine learning, Diagnosis model

## Abstract

**Background:**

Endometriosis (EMs) is an enigmatic disease of yet-unknown pathogenesis. Disulfidptosis, a novel identified form of programmed cell death resulting from disulfide stress, stands a chance of treating diverse ailments. However, the potential roles of disulfidptosis-related genes (DRGs) in EMs remain elusive. This study aims to thoroughly explore the key disulfidptosis genes involved in EMs, and probe novel diagnostic markers and candidate therapeutic compounds from the aspect of disulfidptosis based on bioinformatics analysis, machine learning, and animal experiments.

**Results:**

Enrichment analysis on key module genes and differentially expressed genes (DEGs) of eutopic and ectopic endometrial tissues in EMs suggested that EMs was closely related to disulfidptosis. And then, we obtained 20 and 16 disulfidptosis-related DEGs in eutopic and ectopic endometrial tissue, respectively. The protein-protein interaction (PPI) network revealed complex interactions between genes, and screened nine and ten hub genes in eutopic and ectopic endometrial tissue, respectively. Furthermore, immune infiltration analysis uncovered distinct differences in the immunocyte, human leukocyte antigen (HLA) gene set, and immune checkpoints in the eutopic and ectopic endometrial tissues when compared with health control. Besides, the hub genes mentioned above showed a close correlation with the immune microenvironment of EMs. Furthermore, four machine learning algorithms were applied to screen signature genes in eutopic and ectopic endometrial tissue, including the binary logistic regression (BLR), the least absolute shrinkage and selection operator (LASSO), the support vector machine-recursive feature elimination (SVM-RFE), and the extreme gradient boosting (XGBoost). Model training and hyperparameter tuning were implemented on 80% of the data using a ten-fold cross-validation method, and tested in the testing sets which determined the excellent diagnostic performance of these models by six indicators (Sensitivity, Specificity, Positive Predictive Value, Negative Predictive Value, Accuracy, and Area Under Curve). And seven eutopic signature genes (ACTB, GYS1, IQGAP1, MYH10, NUBPL, SLC7A11, TLN1) and five ectopic signature genes (CAPZB, CD2AP, MYH10, OXSM, PDLIM1) were finally identified based on machine learning. The independent validation dataset also showed high accuracy of the signature genes (IQGAP1, SLC7A11, CD2AP, MYH10, PDLIM1) in predicting EMs. Moreover, we screened 12 specific compounds for EMs based on ectopic signature genes and the pharmacological impact of tretinoin on signature genes was further verified in the ectopic lesion in the EMs murine model.

**Conclusion:**

This study verified a close association between disulfidptosis and EMs based on bioinformatics analysis, machine learning, and animal experiments. Further investigation on the biological mechanism of disulfidptosis in EMs is anticipated to yield novel advancements for searching for potential diagnostic biomarkers and revolutionary therapeutic approaches in EMs.

**Supplementary Information:**

The online version contains supplementary material available at 10.1186/s13036-024-00437-0.

## Background

Endometriosis (EMs) is defined as the ectopic implantation and growth of endometrial-like tissue outside of the uterine cavity, afflicting approximately 5–10% of women of childbearing age worldwide [1, 2]. As a prevalent gynecological disorder associated with dysmenorrhea, chronic pelvic pain, infertility, and depression, EMs poses seriously negative effects on social and psychological functioning [3]. In light of its rising prevalence, frequent recurrence, and significant inadequacies in clinical diagnosis and therapy, endometriosis is unarguably a public health issue and require urgent and close attention. Up to this point, some theories could elucidate the cause of endometriosis, involving the interplay of genetic, immunologic, inflammatory, proangiogenic, and endocrine factors. The precise mechanism of EMs still evades scientific consensus, leading to delayed diagnosis and the extension of beneficial treatment [4]. Thus, probing the potential pathomechanisms, novel diagnostic biomarkers, and screening candidate compounds are crucial to facilitate accurate and timely clinical diagnosis and take into account more comprehensive treatment.

Disulfidptosis is a novel identified form of programmed cell death resulting from disulfide stress [5] .Emerging research suggests that glucose deprivation triggers the dramatic upregulation of solute carrier family 7 member 11 (SLC7A11), which is associated with cystine import and restricts the supply of glucose-derived nicotinamide adenine dinucleotide phosphate (NADPH). And NADPH is an essential reducing agent involved during the process of converting SLC7A11-imported cystine to cysteine. Consequently, heightened absorption of cystine in SLC7A11-high cells contributes to a deleption of intracellular NADPH and an excessive buildup of cystine and other disulfides, which contributes to the formation of abnormal actin cytoskeletal disulfide bonding, ultimately disrupting the actin cytoskeleton and causing cell death [6–8]. Currently, the potential link between disulfidptosis and the onset and progression of various diseases has been extensively investigated, including cancer, liver diseases, and neurological disorders [9–12]. However, the potential involvement of disulfidptosis in the field of gynecological diseases has not been researched and remains an underexplored subject. Thus, thoroughly investigating the role of disulfidptosis in various diseases holds immense significance in broadening up new possibilities for treatment.

EMs, though a non-malignant proliferative condition, exhibits similarities with cancer in terms of the tendency to metastasize, progressive and invasive growth, and estrogen-dependent growth [13, 14]. The retrograde movement of endometrial tissues through the fallopian tubes during menstruation is the most commonly recognized hypothesis to explain the etiology of EMs [15]. In other words, the migration and invasion of refluxed endometrial pieces to locations outside the uterus are necessary for the development of endometrial ectopic lesions necessitate [16]. Thus, cytoskeleton and related proteins tend to exert a prominent effect on regulating cell motility, adhesion, invasion, and proliferation in EMs [17]. Robust evidence exists the expression of the actin-binding protein and alpha-actinin, is elevated in the ectopic endometrium [1]. Additionally, research conducted to identify differential expression genes in EMs reveals that the dysregulation of the actin cytoskeleton could play a key role in the pathogenic mechanism of EMs [18]. It implies a possible connection between disulfidptosis and EMs. Nevertheless, due to the novelty of disulfidptosis, there is still a dearth of relevant researches specifically examining the underlying mechanism of association between them.

**Fig. 1:**
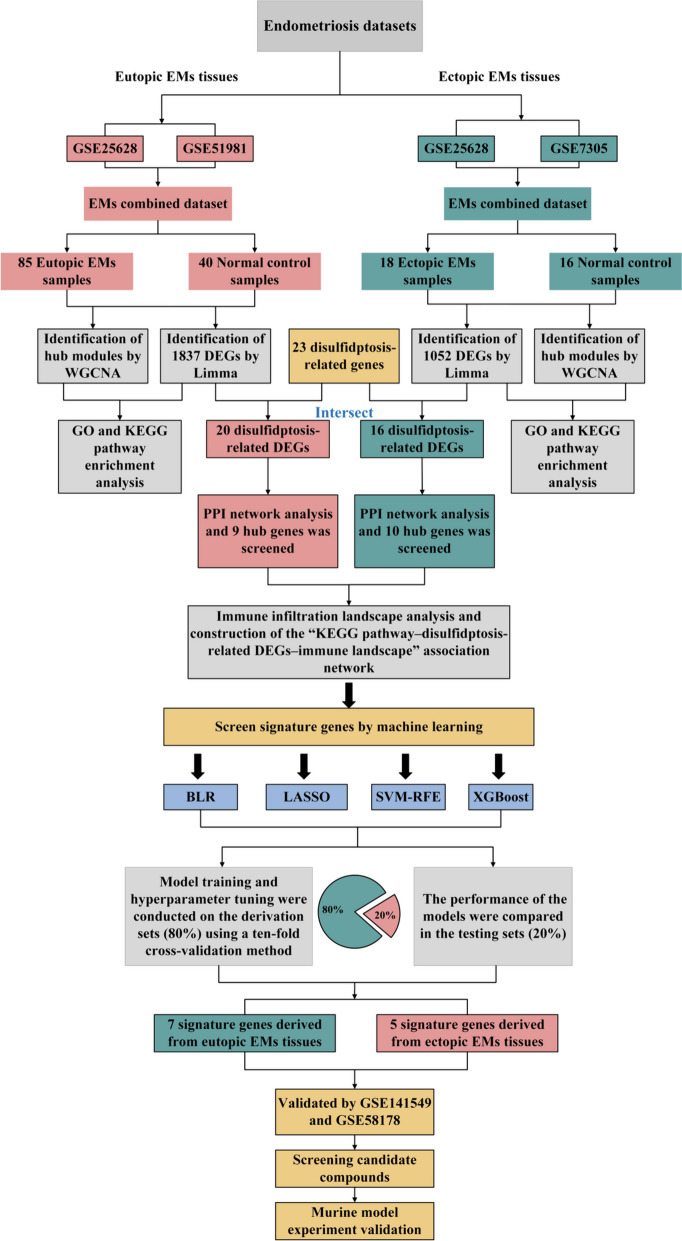
Flow chart for unraveling pathogenesis, biomarkers, and potential therapeutic agents for endometriosis associated with disulfidptosis

In this study, microarrays regarding EMs were sourced from the Gene Expression Omnibus (GEO) database, and disulfidptosis-related genes were acquired from previous studies for comprehensive analysis. Initially, the modules closely related to the eutopic and ectopic endometrial tissues of EMs were identified by weighted gene co-expression network analysis (WGCNA), with enrichment analysis conducted on genes within the critical modules. Afterwards, differentially expressed genes (DEGs) were screened in both eutopic and ectopic endometrial tissues, and the potential molecular mechanisms of EMs in both eutopic and ectopic endometrial tissues were further investigated employing Gene Ontology (GO) and the Kyoto Encyclopedia of Genes and Genomes (KEGG) pathway. Subsequently, the emphasis turned to disulfidptosis-related DEGs, and a protein-protein interaction (PPI) network was established to confirm hub genes. Furthermore, we evaluated the immune infiltration landscape in both eutopic and ectopic endometrial tissue of EMs and analyzed the link between immuocytes, human leukocyte antigen (HLA) gene sets, immune checkpoints, and disulfidptosis-related DEGs. Concerning searching for characteristic biomarkers of EMs, we discerned specific diagnostic biomarkers from disulfidptosis-related DEGs by machine learning and further validated their accuracy with independent validation datasets. Additionally, we screened candidate compounds targeting disulfidptosis-related signature genes, and these compounds could potentially impede the progression of EMs, either backed by empirical evidence or approved by the Food and Drug Administration (FDA). Ultimately, animal experiments were carried out to verify alterations of signature genes linked with EMs and to assess the impact of the drug on these genes and on the pathological features of EMs. The flow chart was displayed in Fig. 1. Collectively, this study first investigates the pathomechanism of EMs in terms of disulfidptosis with in-depth bioinformatics analysis, machine learning, and animal experiments, which offers a novel reference for the diagnosis and therapy of EMs.

## Materials and methods

### Data collection, pre-processing and normalization

In this study, we screened publicly available datasets from the GEO database (https://www.ncbi.nlm.nih.gov/geo/) [19] with the search terms “endometriosis”, “endometrioses”, “endometrioma”, “ovarian endometriosis” and “ovarian endometrioma”. The criteria for filtering the datasets were as follows: 1) Homo sapiens; 2) expression profiling by array; 3) the experiment included patients with EMs and age-matched normal women; 4) the sample size was at least 10 people, with at least 5 participants in each group. Finally, we obtained RNA-seq datasets, including GSE25628, GSE51981, GSE141549, GSE7305, and GSE58178, among which GSE25628, GSE51981, and GSE141549 datasets included 186 eutopic EMs samples and 83 normal samples, GSE25628, GSE7305, and GSE58178 included from 24 ectopic EMs samples and 22 normal samples. In addition, the GSE25628, GSE51981, and GSE7305 datasets were used as the training cohorts, and GSE141549 and GSE58178 datasets were considered as independent validation sets to assess the predict performance of signature genes in eutopic and ectopic endometrium of EMs. The details were available in Table 1. Besides, the disulfidptosis-related genes were obtained from previous studies [6, 20–23]. Subsequently, the ‘affy’ package in R software (version 4.3.0) was applied to conduct multi-array analysis (RMA) background correction, complete log2 transformation, quantile normalization, and median polish algorithm summarization.

**Table 1:** The basic information of included dataset

GSE No.	No.of samples	Platform	Description	Country	Type	Category
GSE25628	6 vs. 8	Affymetrix Human Genome U133A 2.0 Array	Endometrium from 8 EMs patients with eutopic endometrium and 6 normal controls	Italy	training set	eutopic endometrial tissue
GSE51981	34 vs. 77	Affymetrix Human Genome U133 Plus 2.0 Array	Endometrium from 77 EMs patients with eutopic endometrium and 34 normal controls	USA	training set	eutopic endometrial tissue
GSE141549	43 vs. 101	Illumina HumanHT-12 V4.0 expression beadchip	Endometrium from 101 EMs patients with eutopic endometrium and 43 normal controls	Finland	independent validation set	eutopic endometrial tissue
GSE25628	6 vs. 8	Affymetrix Human Genome U133A 2.0 Array	Endometrium from 8 EMs patients with ectopic endometrium and 6 normal controls	Italy	training set	ectopic endometrial tissue
GSE7305	10 vs. 10	Affymetrix Human Genome U133 Plus 2.0 Array	Endometrium from 10 EMs patients with ectopic endometrium and 10 normal controls	USA	training set	ectopic endometrial tissue
GSE58178	6 vs. 6	HumanHT-12 V3.0 expression beadchip	Endometrium from 6 EMs patients with ectopic endometrium and 6 normal controls	USA	independent validation set	ectopic endometrial tissue

### Construction of the co-expression network and hub module identification by WGCNA

The R package “WGCNA” was utilized to construct a gene co-expression network on genes to understand the linkage between them. The “goodSamplesGenes” function was used to perform sample clustering to identify and remove outliers. Subsequently, we established a weighted adjacency matrix and defined a correlation power (soft thresholding parameter) to describe the correlation strength between the nodes. Then, we used average linkage hierarchical clustering and a dynamic tree cut function based on Topological Overlap Measure (TOM) dissimilarity measurement to classify genes showing similar expression profiles with gene modules, each containing at least 30 genes (minModuleSize = 30). Thereafter, we constructed module relationships, calculated the correlation between gene modules and phenotypes, with module eigengenes and module membership to the important modules relevant to clinical traits. Finally, modules with the greatest Spearman correlation coefficient were identified as the significant modules.

### Identification of DEGs

To verify the DEGs in eutopic and ectopic endometrium tissues of EMs, we separately conducted two differential gene analyses on datasets of EMs patients and normal groups, in which one differential gene analysis was between eutopic EMs samples and normal samples, and the other was between ectopic EMs samples and normal samples in the training cohorts. Specifically, DEGs in EMs patients were screened by the R “limma” package with a threshold of *P*-value < 0.05 and |log2 fold change (FC)| >1. Volcano plots and heatmaps depicted the results by the R “ggplot2” package.

### Enrichment analysis on hub module genes and DEGs

To investigate the concrete biological mechanism of hub module genes derived from WGCNA and DEGs of EMs, enrichment analysis was performed by importing these genes into the metascape website (https://metascape.org/gp/index.html) [24]. We carried out KEGG pathway enrichment analysis on hub module genes and GO and KEGG pathway enrichment analysis on DEGs. Terms with *P <* 0.05 were considered significant.

### Screening for disulfidptosis-related DEGs, PPI analysis and correlation analysis

To further figure out the expression profiling pattern of disulfidptosis-related genes in eutopic and ectopic endometrium tissues of EMs, we overlapped the disulfidptosis-related genes with DEGs in eutopic and ectopic endometrium tissues of EMs, separately (*P <* 0.05 and |log2FC)|>0.2, which were designated as the disulfidptosis-related DEGs. Furthermore, we conducted PPI network analysis to investigate the interaction among disulfidptosis-related DEGs by using the STRING database, which contained physical and functional interactions between proteins [25]. Then, hub genes were screened by Cytohubba, which provided 12 algorithms to assess the significance of genes in the PPI network [26]. And the top 10 out of the 12 algorithms were defined as hub genes. Besides, correlation analysis was carried out to explore the mutual effect of disulfidptosis-related DEGs using the pearson correlation coefficient. All results were shown by the R"corrplot" package.

### Tissue localization of disulfidptosis-related DEGs and immune infiltration landscape analysis

We obtained the tissue localization information of disulfidptosis-related DEGs from the BioGPS (http://biogps.org) [27] and the Human Protein Atlas (https://www.proteinatlas.org/) [28]. To better understand the physicochemical properties and functions of the proteins encoded by the hub genes, we searched the BioGPS database, GeneCards (https://www.genecards.org/) [29], Alliance of Genome Resources (https://www.alliancegenome.org/) [30], and UniProt (https://www.uniprot.org/) websites [31]. Besides,the immune infiltration matrix was obtained using the CIBERSORT algorithm by R software. And Wilcoxon test was conducted to compare 22 immunocyte profilings between EMs groups and control groups, and the R “ggplot2” package was utilized to visualize the immune infiltration matrix. Besides, the expression profiling of immune checkpoints and HLA gene sets was quantified. Furthermore, we conducted Spearman correlation analysis between disulfidptosi-associated DEGs and immunocytes as well as immune checkpoints by using the R “corrplot” package.

### Construction of the “KEGG pathway–disulfidptosis-related DEGs–immune landscape” association network

To gain a global comprehension of the biological functions performed by characterized genes and their connection to the immune landscape, “KEGG pathway-disulfidptosis-related DEGs-immune landscape” network was constructed using XTalkDB (http://www.xtalkdb.org/) [32]. First, the cross-talk relationship between the KEGG pathways enriched by disulfidptosis-related DEGs was obtained through the XTalkDB website. Then, we entered the mapping between disulfidptosis-related DEGs and immune cells, HLA molecules, and immune checkpoints into XTalkDB. Finally, the “KEGG pathway-disulfidptosis-related DEGs-immune landscape” association network was shown using Cytoscape 3.7.1 software.

### Construction of disulfidptosis-related diagnosis model based on machine learning

To further conduct the multivariable DEGs selection, we first eliminated the high mean absolute correlation of DEGs by using the correlation matrix method. For each DEGs, the mean absolute correlation based on the pair-wise correlations was calculated. If a pair-wise correlation was > 0.7, the DEGs with greater absolute correlation were removed by using the caret package in R [33, 34]. Machine learning was performed to screen disulfidptosis-related signature genes by the binary logistic regression (BLR), the least absolute shrinkage and selection operator (LASSO), the support vector machine-recursive feature elimination (SVM-RFE), and the extreme gradient boosting (XGBoost) algorithms. BLR can effectively and forcefully evaluate the influence of independent variables on binary outcomes by measuring their specific contributions [35]. The LASSO algorithm, known for its variable screen and regularization features, can extract the best features from large datasets by eliminating relatively insignificant features to improve the accuracy and interpretability of prediction results [36]. SVM-RFE is a vector-supporting machine learning algorithm that can identify and filter irrelevant feature variables, achieving enhanced classification accuracy and performance [37]. And XGBoost is a very effective method for various classification problems. It is an extreme gradient boosting method which can rank features from most to least important [38]. Firstly, BLR was performed to predict signature genes by disulfidptosis-related DEGs in the R “rms” package. Then, the R “glmnet” package was performed to run the LASSO algorithm. Besides, we used “kernlab”, “e1071” and “caret” packages in R software to conduct SVM-RFE. Furthermore, the XGBoost classifier was constructed using the R package “xgboost”. In addition, the datasets were divided into a derivation set and a testing set according to the ratio of 80% and 20%. Model training and hyperparameter tuning were conducted on the derivation set (80%). Specifically, ten-fold cross-validation ensures the stability and generalisation ability of the model by randomly dividing the training set into ten equal parts, training with nine of the data each time and validating with the remaining one, and so on ten times. The optimized models are cross-validated and then evaluated in the testing sets(20%). Accordingly, six key indicators were selected to evaluate the performance of the model, including Sensitivity, Specificity, Positive Predictive Value (PPV), Negative Predictive Value (NPV), Accuracy, and Area Under Curve (AUC). Finally, the overlapped genes in the above algorithms are characterized by Venn diagrams and defined as disulfidptosis-related signature genes. What’s more, we also evaluated the diagnostic performance of signature genes using the receiver operating characteristic (ROC) curve. The AUC>0.7 suggested the prediction was optimal. Then, on the basis of the signature genes, the “rms” R package was developed to establish the nomogram prediction model, in which “points” represents the score of candidate genes and “total points” represents the sum of all the scores of the genes aforementioned. Besides, an ROC curve was performed to determine whether the decision based on the nomogram benefited the diagnosis of EMs. And the calibration curves were constructed to evaluate the predictive efficiency of the nomogram in EMs. More importantly, to ensure the validity and accuracy of novel signature genes, we also validated signature genes derived from machine learning methods in the independent validation datasets of GSE141549 and GSE58178.

### Screening candidate compounds for disulfidptosis-related signature genes in EMs

Potential compounds which could target disulfidptosis-related signature genes in EMs were screened via the following database, including Comparative Toxicogenomics Database (CTD) (http://ctdbase.org/) [39], the Drugbank (https://go.drugbank.com/) [40] and the drug-gene interaction database (DGIdb, www.dgidb.org/) [41]. Compounds may be enlisted if they satisfy the following conditions: (1) drug-gene pairs with interaction score ≥ 0.1; (2) drugs supported by experiment, clinical investigation, or approval by the FDA. What was more, the correspondence relationships between the candidate drugs and the disulfidptosis-related signature genes were visualized using the Cytoscape software.

### Validating the effect of candidate compounds on signature genes in EMs murine models

#### Animals and administration schedule

According to previous studies [42–44], sample power was assessed based on the “resource equation” approach. The calculation formula is as follows:n is the number of animals in per group. DF means the degrees of freedom (DF), and the permissible range of DF for the error term in an analysis of variance (ANOVA) is between 10 to 20. And k represents the number of groups. According to this formula, in the case of setting up 3 groups, each group of mice is 8 mice.

$$\mathrm{n=DF/k+1}$$

Thus, a total of 24 female C57BL/6 mice (6-8weeks) were obtained from the Experimental Animal Center of Zhejiang Chinese Medical University (SYXK(Z) 2021-0012). Experimental mice were housed in ventilated cages with a 12-hour photoperiod and free access to diet. All experimental procedures and animal care were approved by the Institutional Animal Care and Use Committee of the Zhejiang Chinese Medical University (IACUC-202102-01).

During the experimental period, all experimental procedures were carried out in strict accordance with standard randomized, double-blind, placebo-controlled trials to ensure the objectivity of the experimental model and to eliminate unconscious and intentional human influences in the different experimental groups [45]. The group assignment was based on a computer-generated table of random numbers. And the blinding design was achieved by offering the researchers the blinded study drug regimens. Specifically, mice were randomized into three groups with eight mice per group, including the sham-operated group, the EMs group, and the EMs + tretinoin group. We used an endometriosis model in which endometrial tissues from the donor group were intraperitoneally inoculated into the recipient mice. Firstly, the uterine tissue was obtained from the donor group after euthanization, and then was chopped, enzymolised and centrifuged. Subsequently, mice in the EMs group were immediately transplanted with uterine pellets. Meanwhile, sham-operated group mice received a sham operation but not uterine transplantation. As for drug administration, the EMs + tretinoin group was administrated with tretinoin (10 mg/kg), while the sham operation group and the EMs group were given the same dose of normal saline. Then, mice in the sham-operated group, EMs group, and EMs + tretinoin group were euthanized 14 days after modelling, and their endometriums were collected for further experiments.

#### Quantitative real-time polymerase chain reaction (qRT-PCR)

After sufficient grinding, total RNA was extracted from endometrial tissues using the TRIZOL reagent. Then the HiFiScript cDNA synthesis kit (Cat. No: CW2569M, CWBIO) was utilized for reverse transcription. The SYBR Green Pro Taq HS premixed qPCR kit (Cat. No: AG11701, AGBIO) was also employed for the PCR system. The 2−△△CT method was adopted to explore differences of inflammatory factors. The primer sequences were as followed: tumor necrosis factor alpha (TNF-α): FORWARD: ACGCTCTTCTGTCTACTGAACTTCG, REVERSE: TGGTTTGTGAGTGTGAGGGTCTG; interleukin-1beta (IL-1β): FORWARD: CTCGCAGCAGCACATCAACAAG, REVERSE: CCACGG GAAAGACACAGGTAGC.

#### Immunofluorescence staining

Frozen endometrial tissues were sliced to 10µm sections and dried at room temperature for 15 min. The sections were fixed with 4% paraformaldehyde for 30 min, followed by primary antibodies overnight at 4°C: CD31 (1:200, Cat. No: 38365, signalway antibody), vascular endothelial growth factor (VEGF) (1:200, Cat. No: 29301, signalway antibody), MYH10 (1:200, Cat. No: ab230823, abcam), PDLIM1 (1:200, Cat. No: 56823, signalway antibody), CD2AP (1:200, Cat. No: 2135, CST). After being washed three times, the sections were followed by secondary antibodies at room temperature in the dark for 2h: goat anti-rabbit IgG H&L(Alexa Fluor® 488) (1:200, Cat. No: ab150077, abcam). The sections were stained with DAPI (4',6-diamidino-2-phenylindole) and observed using digital pathological section (fluorescence) scanning analyzer (VS120-S6-W, OLYMPUS). The mean immunofluorescence intensity capture and analyze of CD31, VEGF, MYH10, PDLIM1 and CD2AP were performed by Image J software (National Institutes of Health, Bethesda, Maryland).

### Statistical analysis

All data were analyzed by Graphpad Prism 9 (GraphPad Software, San Diego, CA, United States). Normality and variance homogeneity assumptions were confirmed using Shapiro-Wilk's and Brown-forsythe tests, respectively. Next, the normal homogeneous data were submitted to Student’s t test or one-way analysis of variance (ANOVA), followed by Dunnett’s test for the post hoc test if necessary. When heterogeneity of variance was found, an alternative to the classical approach, the Welch’s t-test or Welch’s ANOVA, was applied. The difference was considered statistically significant when *P* < 0.05.

## Results

### Identification of key modules via WGCNA and enrichment analysis

**Fig. 2:**
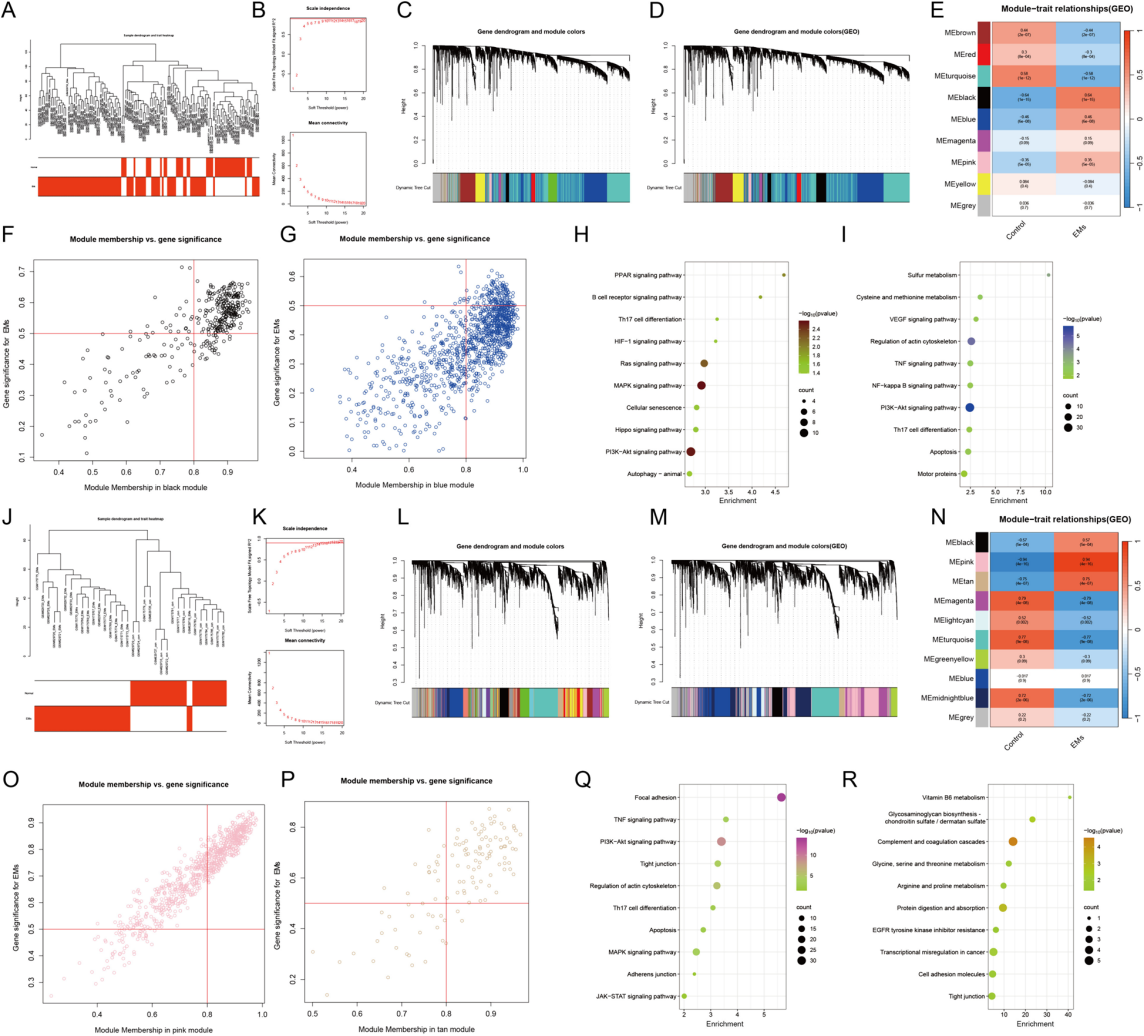
WGCNA based on eutopic and ectopic endometrial tissues, respectively, and enrichment analysis of key modules. **A**, **J** The sample dendrograms and trait heatmaps of all genes. **B**, **K** Scale independence and mean connectivity for the different soft thresholds. **C**-**D**, **L**-**M** The cluster dendrograms of various modules are divided by hierarchical clustering. **E**, **N** The heatmap of different modules with different colors. Red and blue imply positive and negative correlation respectively, and color depth implies degree of correlation. For each module, correlation coefficients and *P* -value are noted. **F**-**G**, **O**-**P** The scatter plots of modules with the highest correlation, including the black module, the blue module, the pink module, and the tan module respectively. **H**-**I**, **Q**-**R** KEGG analysis of modules with the highest correlation presented by the gene ratio (X-axis) and KEGG terms (Y-axis). The circle size represents gene numbers, and the color refers to the *P* -value

WGCNA was applied to divide gene modules in the eutopic endometrial tissues (Fig. 2A-D) and ectopic endometrial tissues (Fig. 2J-M). A total of eight and nine gene co-expression modules were identified by different colours, respectively (Fig. 2E, N). The black module (*r* = 0.64, *P <* 0.05) and the blue module (*r* = 0.46, *P <* 0.05) demonstrated the two highest correlations with eutopic tissues in all modules (Fig. 2E-G). The pink module (*r* = 0.94, *P <* 0.05) and the tan module (*r* = 0.75, *P <* 0.05) likewise showed the two highest correlations with ectopic endometrial tissues (Fig. 2N-P). Thus, these modules were considered as the key modules.

To better understand the biological mechanism, KEGG enrichment analysis was performed (Fig. 2H-I, Q-R). As for the eutopic endometrial tissues, enrichment analysis revealed black module genes were mainly activated in the PPAR signaling pathway, Th17 cell differentiation, and the HIF-1 signaling pathway (Fig. 2H). Genes in the blue module were enriched in sulfur metabolism, cysteine and methionine metabolism, and regulation of actin cytoskeleton (Fig. 2I). In addition, the KEGG enrichment analysis of key module genes of ectopic endometrial tissues revealed that pink module genes were mainly involved in focal adhesion, tight junction, and apoptosis (Fig. 2Q). The tan module genes were activated in complement and coagulation cascades, glycine, serine and threonine metabolism, and tight junction (Fig. 2R).

### DEGs and functional enrichment analysis

**Fig. 3:**
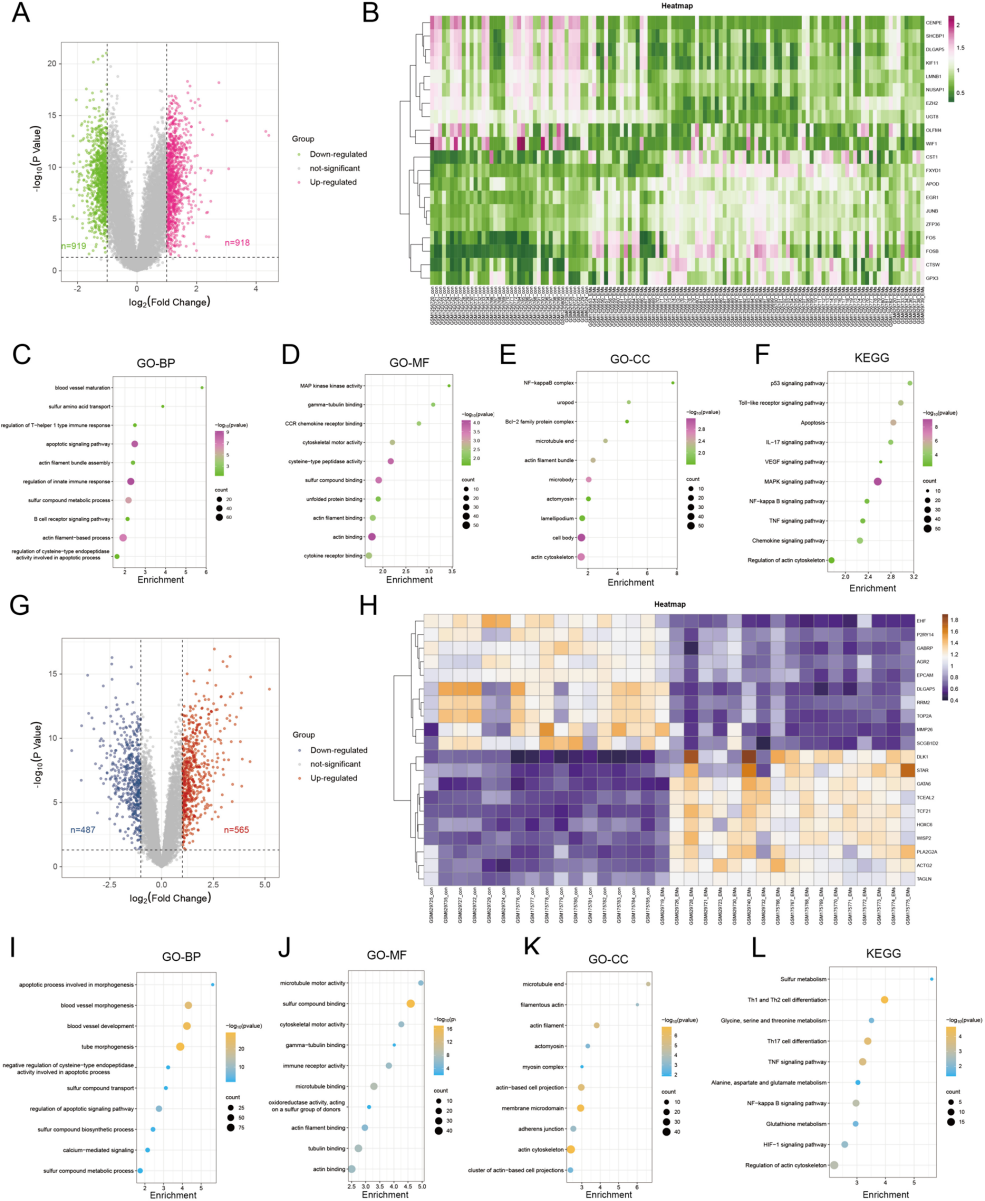
Identification and enrichment analysis of DEGs in eutopic and ectopic endometrial tissues respectively. **A**, **G** The volcano plot depict all DEGs, of which pink and red points represent up-regulated DEGs, and green and blue points represents down-regulated DEGs. **B**, **H** The heatmaps of DEGs presented by the samples of EMs cases or controls (X-axis) and DEGs (Y-axis). **C**-**E**, **I**-**K** The GO analysis of DEGs, including biological process, molecular function, and cellular component respectively. **F**, **L** the KEGG analysis of DEGs. The X-axis represents gene enrichment, and the Y-axis refers to KEGG terms. The circle size represents gene numbers, and the color refers to the *P* -value

A total of 1837 DEGs were identified in eutopic endometrial tissues by using differential gene analysis, among which 918 were up-regulated and 919 were down-regulated (Fig. 3A-B). The Gene Ontology-Biological Process (GO-BP) analysis suggests that DEGs were significantly involved in blood vessel maturation, sulfur amino acid transport, and apoptotic signaling pathway (Fig. 3C). The Gene Ontology-Molecular Function (GO-MF) analysis revealed that the DEGs primarily regulated gamma-tubulin binding, cytoskeletal motor activity, and sulfur compound binding (Fig. 3D). And the Gene Ontology-Cellular Component (GO-CC) analysis showed that proteins expressed by the DEGs were mainly distributed in the NF-kappaB complex, actin filament bundle, and actomyosin (Fig. 3E). In addition, KEGG analysis revealed DEGs in eutopic endometrial tissues were involved in the Toll-like receptor signaling pathway, apoptosis, regulation of actin cytoskeleton (Fig. 3F).

Regarding the ectopic endometrial tissues, there were 1052 DEGs, including 565 up-regulated genes and 487 down-regulated genes (Fig. 3G-H). The significant GO-BP terms were mainly associated with apoptotic process involved in morphogenesis, negative regulation of cysteine-type endopeptidase activity involved in apoptotic process, and sulfur compound transport (Fig. 3I). The GO-MF terms were mainly correlated with microtubule motor activity, sulfur compound binding, and immune receptor activity (Fig. 3J). Besides, the GO-CC terms of these DEGs were significantly related to the microtubule end, filamentous actin, and actomyosin (Fig. 3K). Furthermore, KEGG enrichment analysis was performed, and then DEGs in ectopic endometrial tissues were mainly enriched in sulfur metabolism, Th1 and Th2 cell differentiation, and regulation of actin cytoskeleton (Fig. 3L).

### Identification of disulfidptosis-related DEGs, PPI analysis, and correlation analysis

**Fig. 4:**
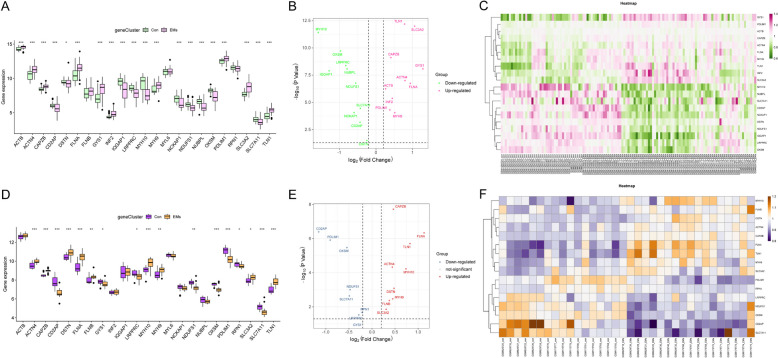
Identification of disulfidptosis-related DEGs in eutopic and ectopic endometrial tissues respectively. **A**, **D** The boxplots depict the expression profile of disulfidptosis-related DEGs between EMs and normal controls. **B**, **E** The volcano plots of disulfidptosis-related DEGs. Pink and red points represents up-regulated disulfidptosis-related DEGs, and green and blue points represents down-regulated disulfidptosis-related DEGs. **C**, **F** The heatmaps of disulfidptosis-related DEGs. Each row represents a disulfidptosis-related DEG, and each column represents a sample. * represents *P<*0.05, ** represents *P<*0.01, and *** represents *P<*0.001 compared with the normal controls

**Fig. 5:**
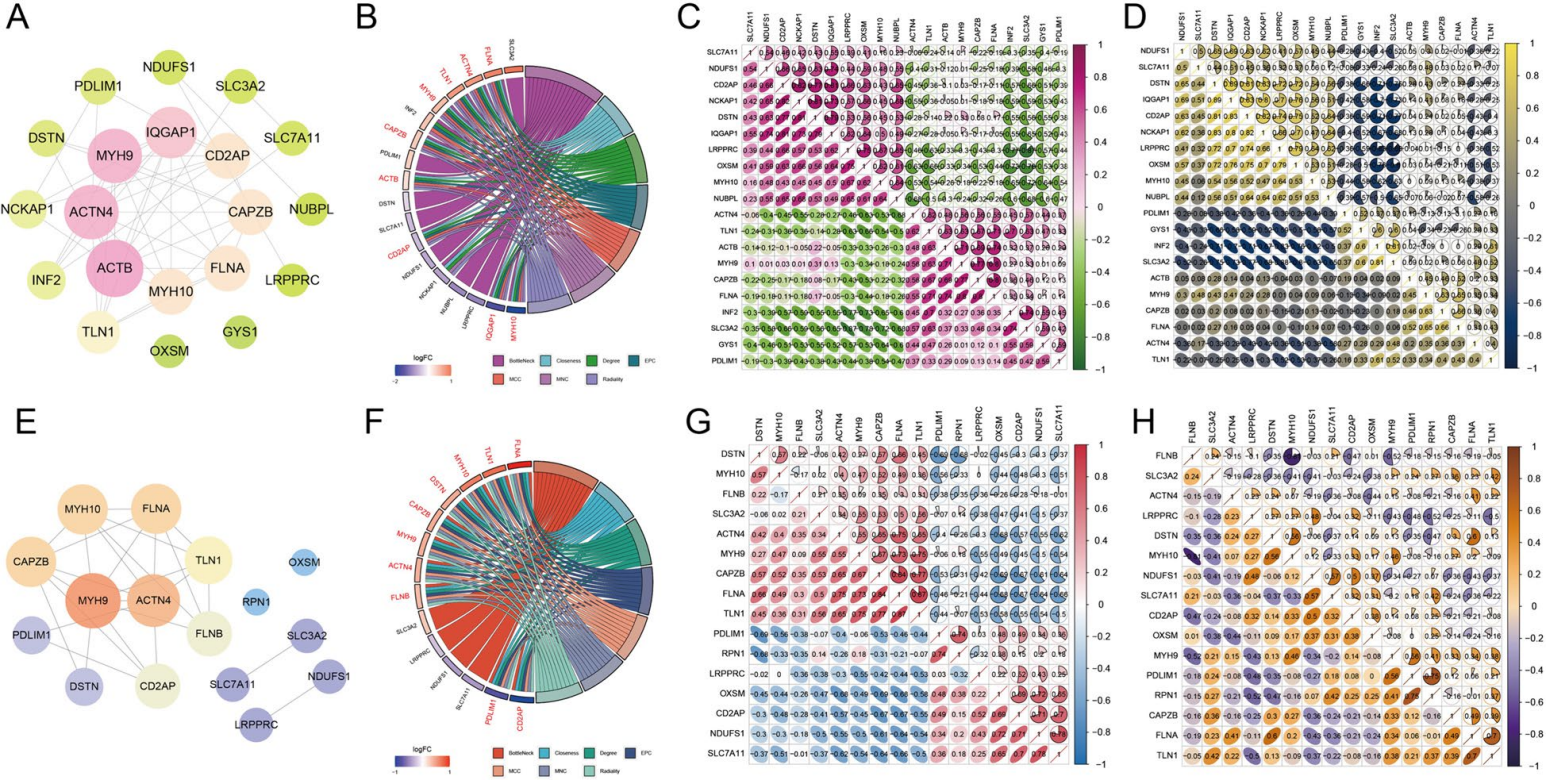
The PPI network and correlation analysis of genes in all samples and in eutopic and ectopic endometrial tissues of EMs, respectively. **A**, **E** The PPI network was composed of disulfidptosis-related DEGs. The circle nodes represent hub genes and edges refer to the interactions between nodes. **B**, **F** The chord diagram shows the hub genes in eutopic and ectopic endometrial tissues of EMs under the MCC algorithm. **C**-**D**, **G**-**H** The correlation heatmaps between disulfidptosis-related DEGs in all samples and in eutopic and ectopic endometrial tissues of EMs, respectively

Considering that the key module genes and DEGs were closely related to sulfur compound metabolism and cell apoptosis, we further investigated disulfidptosis-related genes in EMs. Totally, there were 20 disulfidptosis-related DEGs in eutopic endometrium tissues of EMs, in which ten genes were up-regulated and ten genes were down-regulated (Fig. 4A-C). Then, the PPI network was scrutinized, in which nine hub genes in eutopic endometrium tissues were screened by 12 algorithms using the CytoHubba, including FLNA, ACTN4, TLN1, MYH9, CAPZB, ACTB, CD2AP, IQGAP1, and MYH10 (Fig. 5A-B). In addition, correlation analysis was employed for disulfidptosis-related DEGs in all samples and in EMs samples, respectively. And results showed NCKAP1 was most positively associated with CD2AP (*r* = 0.82, *P* < 0.05), and SLC3A2 was most negatively correlated with LRPPRC (*r* = -0.87, *P* < 0.05) in all samples. Besides, IQGAP1 was most positively associated with DSTN (*r* = 0.89, *P* < 0.05), and SLC3A2 was most negatively correlated with LRPPRC (*r* = -0.88, *P* < 0.05) in eutopic endometrial tissue samples (Fig. 5C-D).

Likewise, 16 disulfidptosis-related DEGs were obtained in ectopic endometrium tissues of EMs, in which nine genes were up-regulated and seven genes were down-regulated (Fig. 4D-F). After that, the PPI network was constructed to reveal their interactions, and the hub genes of ectopic endometrial tissues were also screened as mentioned above, including FLNA, TLN1, MYH10, DSTN, CAPZB, ACTN4, MYH9, FLNB, CD2AP, and PDLIM1 (Fig. 5E-F). Furthermore, in all samples, the correlation analysis suggested a positive relationship between TLN1 and FLNA (*r* = 0.87, *P* < 0.05), and a remarkably negative correlation between PDLIM1 and DSTN (*r* = -0.69, *P* < 0.05), as well as OXSM and CAPZB (*r* = -0.69, *P <* 0.05). Besides, in ectopic endometrial tissues, RPN1 showed the most positive association with PDLIM1 (*r* = 0.75, *P* < 0.05), and MYH10 showed the most negative correlation with FLNB (*r* = -0.81, *P <* 0.05) (Fig. 5G-H).

### Tissue location of disulfidptosis-related DEGs and its association with the immune infiltration landscape of EMs

**Fig. 6:**
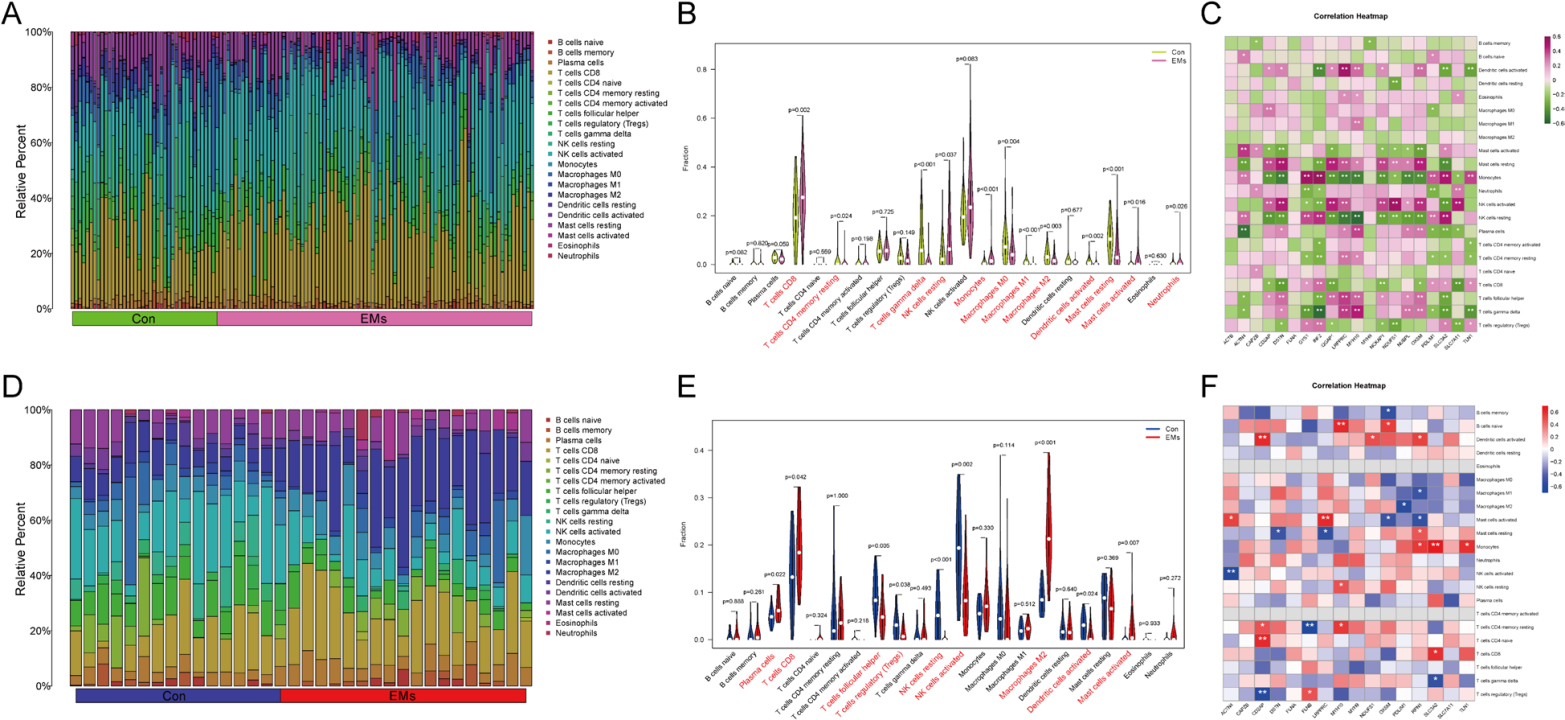
Immune infiltration landscape of EMs and the association of immunocytes with disulfidptosis-related DEGs in eutopic and ectopic endometrial tissues, respectively. **A**, **D** The fraction of 22 subsets of immunocytes in EMs. X axis: each sample; Y axis: relative percentage of each kind of immunocyte. **B**, **E** The violin graphs depict the difference in immune infiltration between EMs (eutopic or ectopic endometrial tissues) and normal controls. **C**, **F** The correlation heatmaps between disulfidptosis-related DEGs and immunocytes

To uncover the detailed localization and functions of proteins from disulfidptosis-related DEGs, we utilized the Human Protein Atlas, the BioGPS database, GeneCards, the Alliance of Genome Resources, and the UniProt website. The details were shown in Supplement Table 1. It’s not too difficult to spot the expression of multiple genes in the uterine tissue and immunocytes, which makes it possible to involve regulating immunization activities in EMs. Thus, we further appreciate the immune landscape of EMs by analyzing changes in immuocytes, HLA gene sets, and immune checkpoints between EMs patients and health controls, and their association with disulfidptosis-related DEGs. As for the quantified level of immunocytes (CIBERSORT), the results showed that five types of immunocytes in eutopic endometrial tissues were remarkably increased when compared to the control group, including CD8 + T cells, resting NK cells, monocytes, activated mast cells, and neutrophils. And seven kinds of immunocytes were decreased, including resting memory CD4 + T cells, gamma delta T cells, M0 macrophages, M1 macrophages, M2 macrophages, activated dendritic cells, and resting mast cells (Fig. 6A-B). Correlation analysis revealed that INF2 had a positive and negative correlation with monocytes (*r* = 0.56, *P <* 0.05) and gamma delta T cells (*r* = -0.56, *P <* 0.05), respectively (Fig. 6C). Likewise, the results in ectopic endometrial tissues revealed that four immunocytes were significantly elevated, including plasma cells, CD8 + T cells, M2 macrophages, and activated mast cells. And five kinds of immunocytes were decreased, including follicular helper T cells, regulatory T cells (Treg cells), resting NK cells, activated NK cells, and activated dendritic cells (Fig. 6D-E). Besides, the correlation analysis revealed that in the ectopic endometrial tissues of EMs, SLC3A2 was most positively associated with monocytes (*r* = 0.68, *P <* 0.05), and FLNB showed the most negative correlation with resting CD4 memory T cells (*r*=-0.67, *P <* 0.05) (Fig. 6F).

**Fig. 7:**
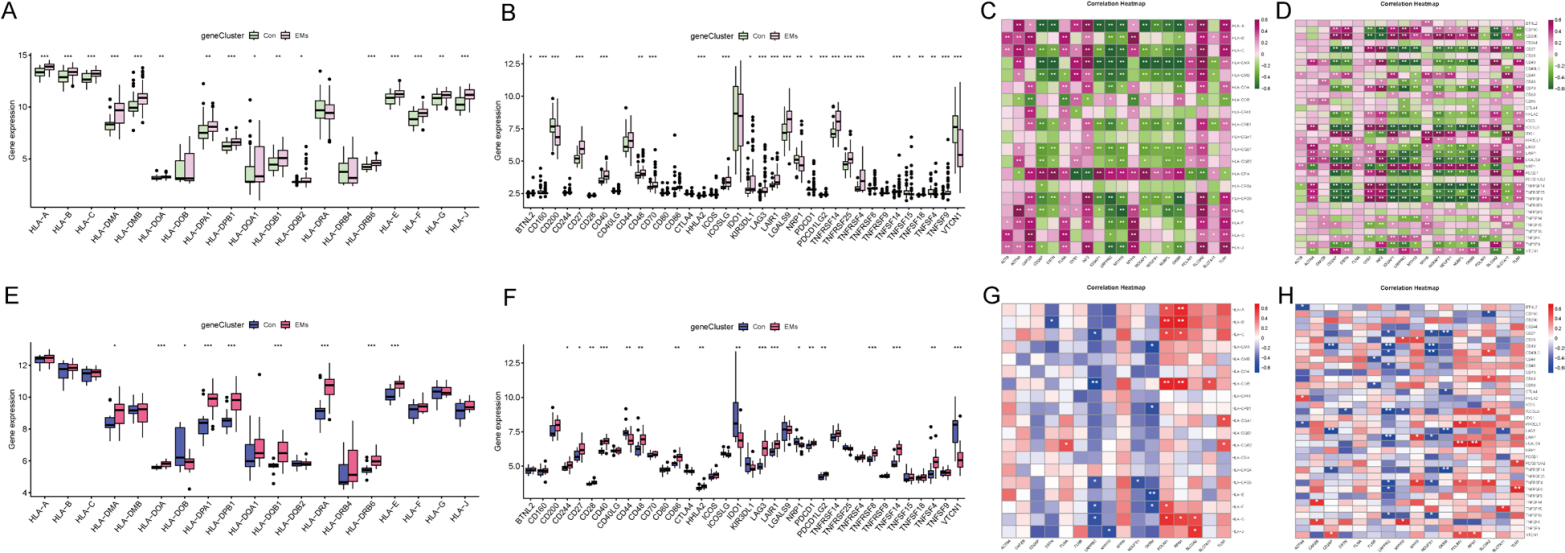
The difference and correlation between HLA gene sets and immune checkpoints in eutopic and ectopic endometrial tissues, respectively. The boxplots depict different gene expressions of HLA gene sets (**A**, **E**) and immune checkpoints (**B**, **F**). **C**, **G** The correlation heatmaps between disulfidptosis-related DEGs and HLA gene sets. **D**, **H** The correlation heatmaps between disulfidptosis-related DEGs and immune checkpoints. * represents *P*  < 0.05, ** represents *P*  < 0.01, and *** represents *P*  < 0.001 compared with the normal controls

Regarding the HLA gene sets and immune checkpoints, both eutopic and ectopic endometrial tissue of patients showed significant variation when compared to normal women (Fig. 7A-B, E-F). Furthermore, we confirmed the correlation of disulfidptosis-related genes with HLA gene sets and immune checkpoints, respectively. For HLA gene sets, SLC3A2 was most positively associated with HLA-A (*r* = 0.84, *P <* 0.05), and LRPPRC showed the most negative correlation with HLA-DMA (*r* = -0.77, *P <* 0.05) from eutopic endometrial tissues (Fig. 7C). HLA-B was most positively associated with RPN1 (*r* = 0.65, *P <* 0.05), and HLA-DOB showed the most negative correlation with LRPPRC (*r* = -0.72, *P <* 0.05) from ectopic endometrial tissues (Fig. 7G). For immune checkpoints, SLC3A2 was most positively associated with TNFRSF4 (*r* = 0.85, *P <* 0.05), and DSTN showed the most negative correlation with TNFRSF4 (*r* = -0.85, *P <* 0.05) from eutopic endometrial tissues (Fig. 7D). LGALS9 was most positively associated with RPN1 (*r* = 0.68, *P <* 0.05), and CD40LG showed the most negative correlation with NDUFS1 (*r* = -0.75, *P <* 0.05) from ectopic endometrial tissues (Fig. 7H).

**Fig. 8:**
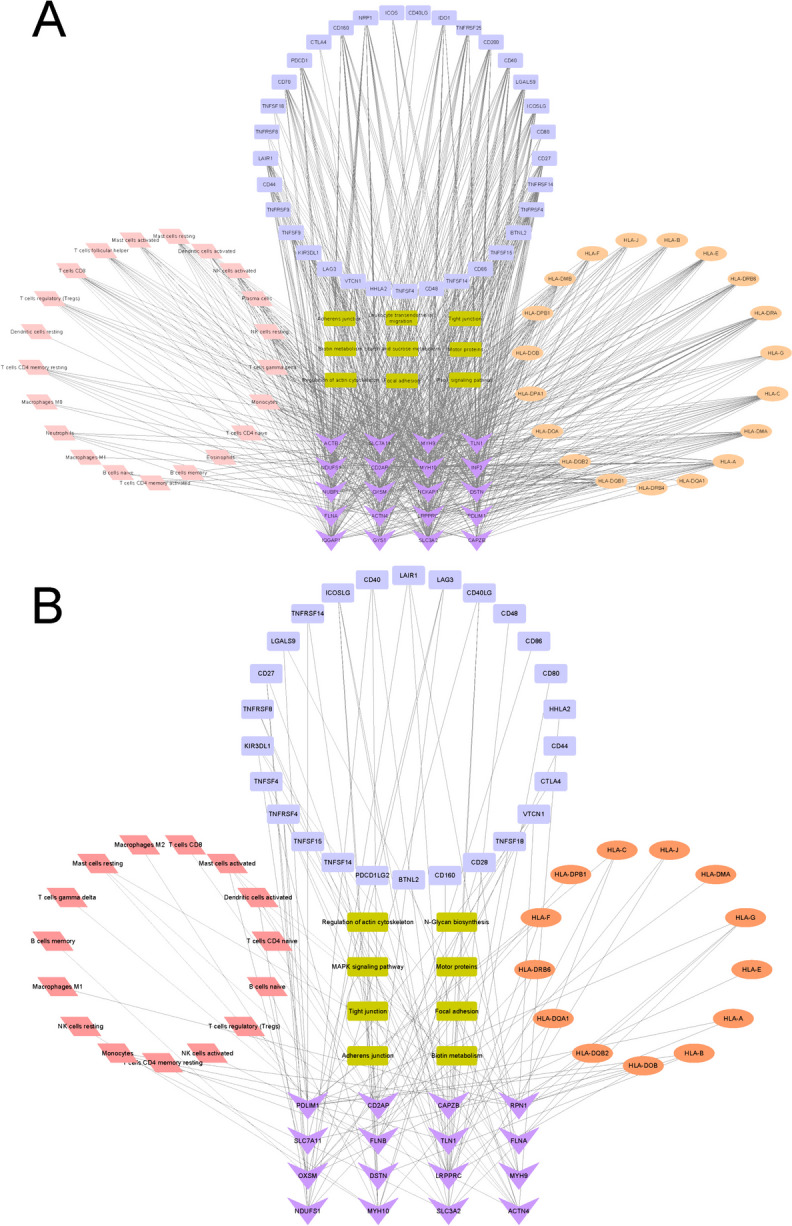
The association network of the “KEGG pathway-disulfidptosis-related DEGs-immune landscape” in eutopic and ectopic endometrial tissues. Different colors represent different terms. Green nodes: the KEGG pathway. Purple nodes: disulfidptosis-related DEGs. Pink nodes: immunocytes. Orange nodes: HLA-related genes. Blue nodes: immune checkpoint-related genes. The black lines refer to the association between nodes

Furthermore, to systematically and intuitively comprehensively examine the association between disulfidptosis-related DEGs and the immune landscape in EMs, we visualized the association of the immune immunoinfiltration landscape (immunocytes, HLA molecules, and immune checkpoints) and the KEGG pathway with the 20 disulfidptosis-related DEGs in eutopic endometrial tissue and the 16 disulfidptosis-related DEGs in ectopic endometrial tissue, respectively, as shown in Fig. 8A and B.

### Screening disulfidptosis-related signature genes of EMs by machine learning

**Fig. 9:**
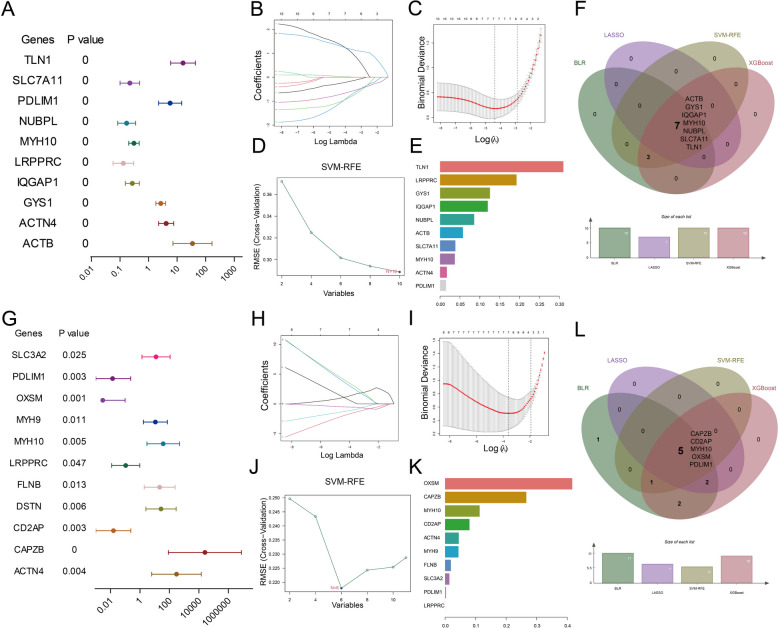
Screening signature genes for EMs in eutopic and ectopic endometrial tissues, respectively. **A**, **G** BLR on eutopic and ectopic disulfidptosis-related DEGs. **B**-**C**, **H**-**I** Screening signature genes by the LASSO algorithm. **D**, **J** Screening signature genes by the SVM-RFE algorithm. **E**, **K** Screening signature genes by the XGBoost algorithm. **F**, **L** The Venn diagrams of intersected genes from BLR, LASSO, SVM-RFE and XGBoost algorithms. The corresponding names and numbers of genes are noted on the figures

Since several of the supervised machine learning approaches could not account for the multicollinearity, we removed the DEGs that were highly correlated with each other. Accordingly, 10 eutopic disulfidptosis-related DEGs and 11 ectopic disulfidptosis-related DEGs were retained (Supplement Fig. 1). Subsequently, four different machine learning methods (BLR, LASSO, SVM-RFE, and XGBoost) were performed to select genes most relevant to the diagnosis and construct diagnostic model. Accordingly, BLR was conducted, and the results revealed that 10 disulfidptosis-related DEGs were independent factors in the eutopic endometrial tissues (Fig. 9A). Then, we obtained 7, 10, and 10 signature genes in the eutopic endometrial tissues through LASSO, SVM-RFE, and XGBoost, respectively (Fig. 9B-E). And 7 signature genes were screened by overlapping the results of the four algorithms, namely ACTB, GYS1, IQGAP1, MYH10, NUBPL, SLC7A11, and TLN1 (Fig. 9F). Likewise, 11 ectopic disulfidptosis-related signature genes were selected by BLR, 7 genes by LASSO, 6 genes by SVM-RFE, and 10 genes by XGBoost, respectively (Fig. 9G-K). And 5 overlapped signature genes were obtained in ectopic endometrial tissues after the intersection of the results of four algorithms, including CAPZB, CD2AP, MYH10, OXSM, and PDLIM1 (Fig. 9L).

**Fig. 10:**
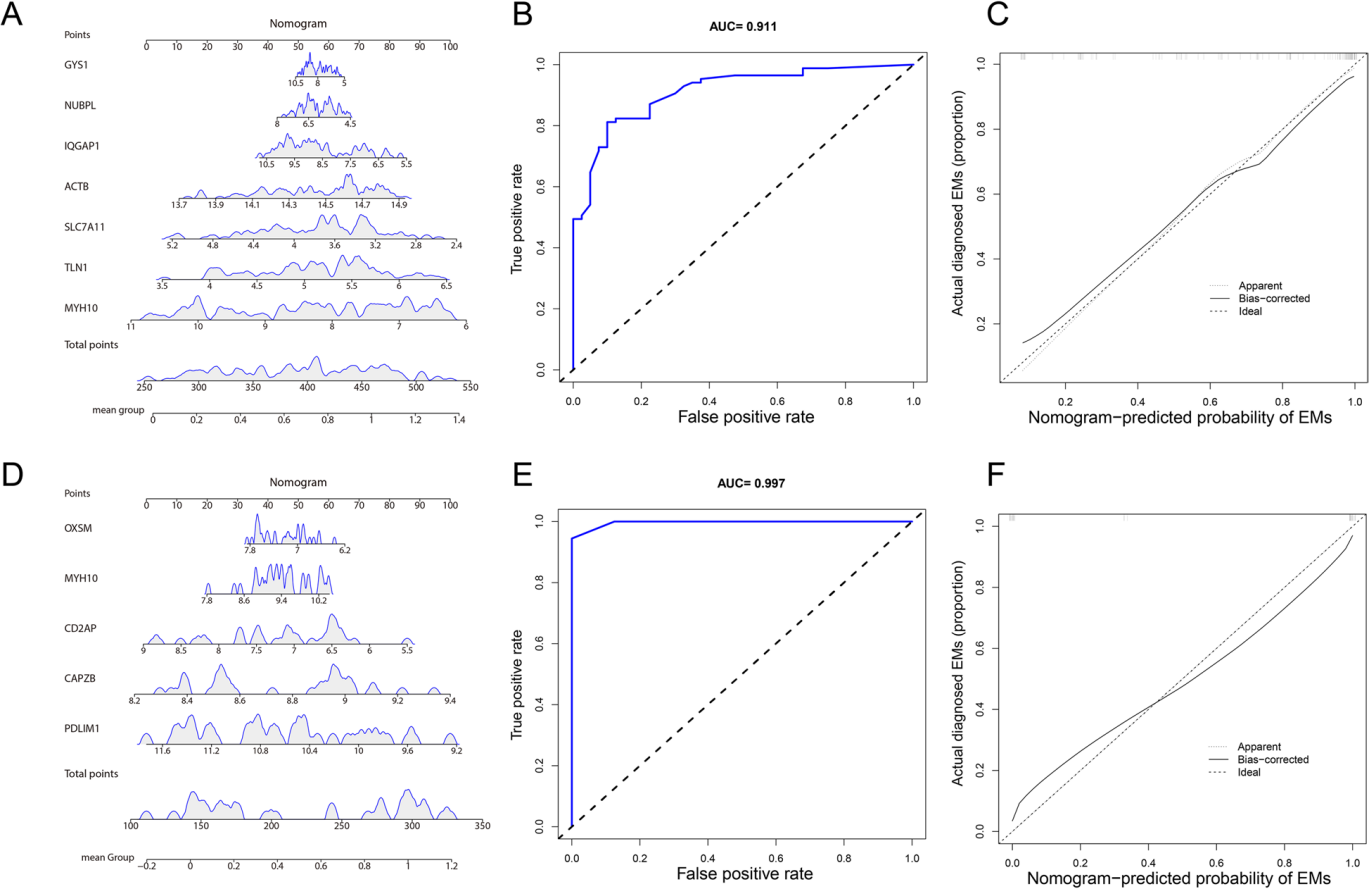
The nomogram model was constructed based on the signature genes of eutopic and ectopic endometrial tissues. **A**, **D** The nomogram prediction model. **B**, **E** The ROC for evaluating the discriminative performance of the nomogram predictive model. **C**, **F** The visual calibration plot for the internal validation of the prediction model

To compare the performance of each feature selection method in predicting endometriosis, we evaluated how each model performed as a classifier on the testing set, as shown in Table 2 and Supplement Fig. 2. These measures include sensitivity, specificity, positive predictive value, negative predictive value, accuracy, and AUC. By comparing these performance indicators, we can comprehensively evaluate the performance of each model in predicting endometriosis. Based on these results, the optimal predictive model can be identified to support clinical decision-making. Moreover, we further focused on the overlapping genes selected by four feature selection methods. Accordingly, the ROC curve was constructed, and the AUC of eutopic signature genes and ectopic signature genes ranged from 0.738 to 0.843 and 0.882 to 0.955, respectively (Supplement Fig. 3), implying excellent diagnostic performance for all signature genes. For better performance in the prediction of EMs, the above signature genes of eutopic endometrial tissues and ectopic endometrial tissues were combined to construct the nomogram model (Fig. 10A, D). The ROC curve was utilized to ascertain the particularity and sensitivity of the nomogram model in diagnosing EMs. The data revealed a greater AUC value for the nomogram compared to each signature gene, implying that the nomogram might hold a robust diagnostic utility for EMs (Fig. 10B, E). The calibration curves displayed that the predictive value of the established nomogram diagnostic model closely matched the performance of the ideal model (Fig. 10C, F), demonstrating a good predictive value in EMs.

**Table 2:** The predictive performance of machine learning models on testing set

In eutopic endometrial tissue						
Model	Sensitivity	Specificity	PPV	NPV	Accuracy	AUC
Binary Logistic Regression	0.75	0.765	0.6	0.867	0.76	0.809
LASSO	0.75	0.765	0.6	0.867	0.76	0.846
SVM-RFE	0.75	0.882	0.75	0.882	0.84	0.816
XGBoost	0.625	0.824	0.625	0.824	0.76	0.846
In ectopic endometrial tissue						
Model	Sensitivity	Specificity	PPV	NPV	Accuracy	AUC
Binary Logistic Regression	1	0.667	0.75	1	0.833	0.889
LASSO	0.667	1	1	0.75	0.833	1
SVM-RFE	0.667	1	1	0.75	0.833	1
XGBoost	0.667	0.667	0.667	0.667	0.667	0.778

**Fig. 11:**
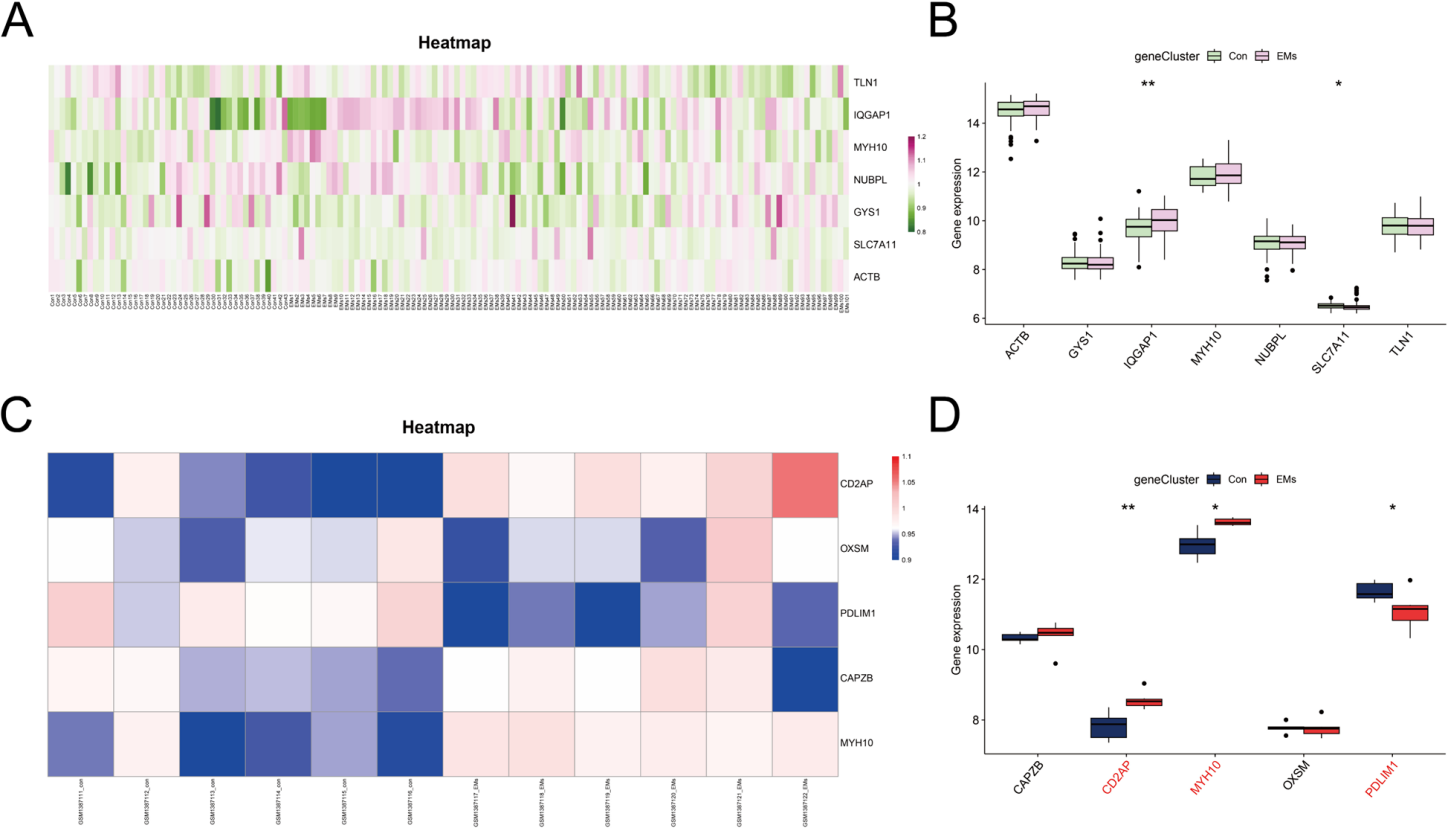
Validation of signature genes for eutopic and ectopic endometrial tissues by independent validation datasets. **A**, **C** The heatmaps revealed the expression profile of eutopic and ectopic signature genes in the independent validation datasets. **B**, **D** The boxplots depict different expression profile of eutopic and ectopic signature genes in the independent validation datasets. * represents *P* <  0.05, ** represents *P* <  0.01 compared with the normal controls

Despite having validated the model's diagnostic ability using ten-fold cross-validation and assessed it in the testing sets (20% datasets), we separately downloaded two additional datasets (GSE141549 and GSE58178) from the GEO database as independent validation datasets to further confirm the accuracy of these diagnostic genes. As for the eutopic signature genes of EMs, the expression changes of IQGAP1 and SLC7A11 were statistically different in the GSE141549 (Fig. 11A-B). Regarding the ectopic signature genes of EMs, the variation in the levels of CD2AP, MYH10 and PDLIM1 were statistically different in the GSE58178 (Fig. 11C-D). These genes are expected to serve as novel markers for the diagnosis of EMs in eutopic and ectopic tissues, respectively, but need to be further validated in prospective large sample studies.

### Screening candidate compounds for disulfidptosis-related signature genes in ectopic endometrial tissues of EMs

To identify potential therapeutic compounds targeting these signature genes in EMs, a screening was conducted using CTD, Drugbank, and DGIdb. After a thorough inquiry, 12 compounds were identified as candidate pharmacological therapeutic agents for the EMs treatment, including cisplatin, cyclosporine, estradiol, ethinyl estradiol, indomethacin, panobinostat, tretinoin, valproic acid, acetaminophen, doxorubicin, phenobarbital, and prednisone (Supplement Fig. 4). As confirmed by relevant studies [46], tretinoin exhibited an effective pharmacological impact on EMs and was expected to be the most potential therapeutic drug for EMs.

### Murine model experiment validation for the efficacy of candidate compounds for EMs

**Fig. 12:**
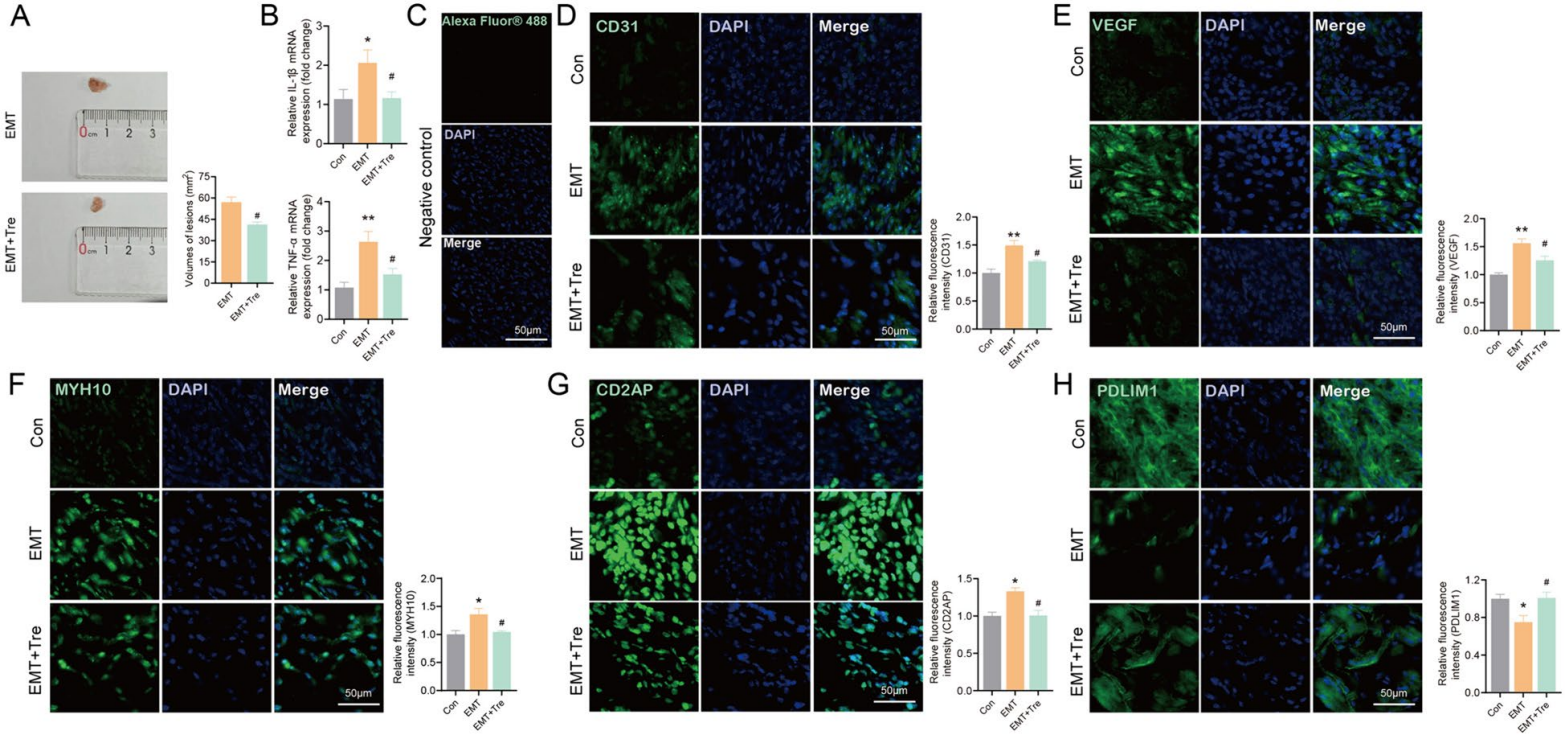
Validation for the theraputic effects of tretinoin in the EMs murine model. **A** The average volume of endometriotic lesions per mouse in the EMT group and the tretinoin-treated group. **B** The relative mRNA expression levels of IL-1β and TNF-α in the control group, the EMT group, and the tretinoin-treated group were quantified by qRT-PCR. **C** Immunofluorescence staining of secondary antibody was used as negative control. **D**-**H** Immunofluorescent staining images of CD31, VEGF, CD2AP, MYH10, and PDLIM1 (green) in the mouse endometrial tissues (control group, EMT group, and EMT + tretinoin group). Nuclei are counterstained with DAPI (blue). The relative fluorescence intensity of CD31, VEGF, CD2AP, MYH10, and PDLIM1 in the control group, the EMT group, and the EMT + tretinoin group was quantified, respectively. * represents *P*  < 0.05 and ** represents *P*  < 0.01 compared with the controls. # represents *P*  < 0.05 compared with the EMT group. Error bars represent the mean ± standard error of mean (SEM). Scale bar, 50µm

The EMs murine model was established to further explore the therapeutic effect of tretinoin for EMs. Student’s t test or one-way ANOVA were conducted due to all the data were normally distributed and homogeneity of variance (Supplement Table 2). The results revealed that the average volumes of lesions per mouse in the tretinoin-treated group were significantly decreased compared to the EMT group (*P <* 0.05) (Fig. 12A). Furthermore, the level of IL-1β was lower in the tretinoin-treated group compared to the EMT group (*P <* 0.05). Meanwhile, the tretinoin-treated group exhibited a noteworthy decrease of TNF-α versus the EMT group (*P <* 0.05) (Fig. 12B). Moreover, the immunofluorescence staining revealed a decreased fluorescence intensity of CD31 and VEGF in the tretinoin-treated group compared to the EMT group (*P <* 0.05) (Fig. 12C-E). More importantly, the effects of tretinoin on disulfidptosis-related signature genes were also observed by immunofluorescence staining. In comparison to the EMT group, tretinoin group showed a substantial decrease of MYH10 and CD2AP (*P* < 0.05), along with a notable elevation of PDLIM1 (*P* < 0.05) (Fig. 12F-H).

## Discussion

Despite numerous investigations and significant endeavors in recent decades, the underlying causes of EMs remain inadequately comprehended. Thus, this current study utilized bioinformatics analysis to comprehensively interpret transcriptome data for eutopic and ectopic endometrial tissue samples of individuals with EMs in the GEO database from a novel viewpoint. Here, we initially explored the engagement of disulfidptosis in the underlying pathomechanism of EMs. The WGCNA method and KEGG analysis depicted genes present in the hub modules of eutopic and ectopic endometrial tissue mostly influnced cell fate, actin cytoskeleton, and sulfur metabolism. Then, through differential expression analysis and enrichment analysis, we reconfirmed implications of DEGs in terms of regulating actin cytoskeleton and sulfur metabolism and identified distinct biological mechanisms of DEGs in ectopic endometrial tissue regarding modulating immune inflammation, angiogenesis, and apoptosis compared to the DEGs in eutopic endometrial tissue. Following that, disulfidptosis-related DEGs were screened in both eutopic and ectopic endometrial tissue, among which nine hub genes in eutopic endometrial tissue and ten hub genes in ectopic endometrial tissue both cooperatively or antagonistically regulate actin cytoskeletons, cellular motility, angiogenesis, and cell proliferation, thereby impacting the progression of EMs. These findings offered initial evidence supporting our conjecture that the pathomechanism of EMs was associated with disulfidptosis. After that, we disclosed the immune infiltration landscape in both eutopic and ectopic endometrial tissue and evaluated the potential correlation between immuocytes, HLA gene sets, immune checkpoints, and disulfidptosis-related DEGs. Essentially, probing these correlations helped to grasp the implication of disulfidptosis on the aberrant immune inflammation in EMs. Furthermore, seven disulfidptosis-related signature genes in eutopic endometria tissues and five signature genes in ectopic endometria tissues were identified via machine learning. The dignostic value of IQGAP1 and SLC7A11 in eutopic endometrial tissues, as well as CD2AP, MYH10, and PDLIM1 in ectopic endometrial tissues, was validated by independent validation datasets. Finally, with a focus on the three signature genes in ectopic endometrial tissues, we screened twelve different compounds and verified the pharmacological impact of tretinoin on EMs and disulfidptosis-related genes in the EMs murine model. Collectively, our research findings could serve as novel targets and insights for further research in this domain.

Through the WGCNA and KEGG analysis, we identified the critical module genes of eutopic and ectopic endometrial tissue of EMs primarily influenced the PI3K-Akt signaling pathway, regulation of the actin cytoskeleton, and sulfur metabolism. The phosphatidylinositol 3-kinase (PI3K)-protein kinase B (AKT) pathway is widely recognized for its crucial role in governing cell growth, invasion, proliferation, and apoptosis [2, 47]. In line with our findings, prior research confirmed elevated levels of phosphorylated AKT (pAKT) in both eutopic and ectopic endometrial cells [48, 49]. Excessive activation of PI3K-AKT can facilitate cell growth and proliferation in eutopic and ectopic tissues, thus promoting the progression [50]. Furthermore, the PI3K-Akt pathway is closely linked to the reconstruction of the actin cytoskeleton [51], which serves as the foundation for celluar molitity, adhesion, invasion, and proliferation [16]. This is essential for forming ectopic lesions. Collectively, these findings support that aberrant activation of PI3K-AKT signaling contributes to disease by affecting cell fate and the cytoskeleton. Beyond that, enrichment analysis also showed critical module genes were involved in the regulation of the actin cytoskeleton and sulfur metabolism. Interestingly, disulfidptosis, a novel programmed death, is regulated by disruption of the actin cytoskeleton attributes to sulfur-containing amino acid metabolism disorder [5]. Current studies have shown that disulfidptosis may affect tumor migration and invasion through the high expression of SLC7A11 [52]. Notably, EMs also exhibits tumor-like features to some extent in terms of the invasive and migratory properties of endometrial tissue presented in ectopic lesions. Collectively, these findings provide vital clues suggesting that disulfidptosis may be closely implicated in the development of EMs.

In addition, we also comprehensively analyzed DEGs in the eutopic and ectopic endometria of EMs. Results displayed that DEGs in both situ and ectopic tissues are strongly associated with immune inflammation. The difference was DEGs in eutopic endometrial tissue were focused on the involvement of intrinsic immunity (activation of the NF-kappaB complex and the Toll-like receptor signaling pathway) and angiogenesis. Whereas the DEGs in ectopic endometrial tissues were more likely to be associated with adaptive immune homeostasis, such as T helper type 1 (Th1)/T helper type 2 (Th2), T helper type 17 (Th17) cell differentiation. This is consistent with the pathologic features of the disease. In terms of ectopic endometrial tissue, endometrial cells relocate to the ectopic site and subsequently stimulate neovascularization so as to ensure sufficient delivery of vital nutrients for lesion survival and proliferation [53]. Echoing the finding mentioned above, the favourable connection between highly vascularized endometriotic tissue and its enhanced proliferation has been confirmed by Miller JE et al. [54]. Thus, the above evidence also supports our findings that DEGs in ectopic tissues are closely involved in the biological process of angiogenesis. Besides, as a type of "autotransplant," the attachment and invasion of endometrial tissue in ectopic lesion mostly rely on proinflammatory cytokines, cell adhesion molecules, and growth factors secreted from elevated macrophages. And the NF-kappaB pathway and Toll-like receptors (TLRs) mainly account for the stimulation of macrophages [55] by secreting TNF-α, interleukin 8 (IL-8), and VEGF [17, 56]. These factors are linked to the occurrence of EMs by facilitating the attachment, growth, and angiogenesis of endometrial cells [57, 58]. Furthermore, continued adherence and infiltration of endometrial cells can inevitably lead to an enduring and excessive inflammatory condition, which can in turn attribute to fibrosis and adhesions of the pelvic tissue in women with EMs [55]. Consistent with our research, a study by Ahn SH et al. also confirmed that ectopic endometrial deposits exhibited increased expression of genes related to the activation of the immune response in comparison to the eutopic endometrium of the same patients, such as tumor necrosis factor (TNF) superfamily genes, HLA genes, and genes that encoded cell adhesion molecules, including intercellular adhesion molecule-1 (ICAM-1) and vascular cellular adhesion molecule-1 (VCAM-1) [59]. Taken together, immune inflammation is a necessary factor for the onset and progression of EMs. In addition, to our surprise, consistent with the function of key module genes, DEGs in both the in situ and ectopic endothelial tissues of EMs also demonstrated a close correlation with sulfur compound metabolism and apoptosis. These findings support the focus of our study of EMs on disulfidptosis. Furthermore, the current studies also unraveled a potential link between disulfidptosis and abnormal immune inflammation [60]. Therefore, this study explored the mechanistic links between disulfidptosis and EMs, and probes the association of disulfidptosis-related DEGs with the immune landscape of EMs.

Considering the potential involvement of disulfidptosis in EMs, we further screened disulfidptosis-related DEGs and their mutual interactions in both eutopic and ectopic endometrial tissues. Subsequently, 9 hub genes in eutopic endometria tissue and 10 hub genes in ectopic endometrial tissue were identified, among which FLNA, ACTN4, TLN1, MYH9, and CAPZB were synergistically upregulated and showed a high degree of correlation in both situ and ectopic tissues. As prior research had described, these up-regulated genes shared the ability to form actin cytoskeletons and promote cellular migration, which might be the key mechanism by which the aforementioned molecules promote the progression of EMs [61–64]. Take TLN1 as an example. Talin1 (TLN1), a cytoskeletal linker protein, is originally identified as an oncogene involved in cellular adhesion, invasion, proliferation, and metastasis [65, 66]. Studies have confirmed that an elevated level of TLN1 in EMs could increase the expression of integrin β3, E-cadherin, N-cadherin, and matrix metalloproteinase-2 (MMP-2), consequently promoting the migration, attachment, and infiltration of endometrial tissue in the abnormal sites [67]. Furthermore, TLN1 has been acknowledged as one of the biomarkers of disulfidptosis in some cancers because it could encode actin-related proteins with disulfide bonds elevated in response to glucose scarcity [68]. Hence, these findings may offer a new insight into the involvement of TLN1-associated disulfidptosis in EMs. Remarkably, FLNA displays striking resemblances to TLN1 in terms of forming the cytoskeleton, interacting with β integrin, and activating AKT/ERK (protein kinase B/extracellular signal-regulated kinase) signals [69], all of which promote the progression by increasing cell survival, angiogenesis, and proliferation [50]. Collectively, these findings provide robust support for the highly synergistic correlation between FLNA and TLN1 in EMs revealed by our study. As for FLNA and MYH9, they serve as substrates for the mechanistic target of rapamycin complex 2 (mTORC2), which governs cell metastasis, proliferation, and neovascularization [70]. Consistently, mTORC2 shows a notable upsurge in EMs, which suggests the participation of FLNA and MYH9 in the ectopic lesion growth [71]. Collectively, in accordance with our study, these findings showed the substantial synergistic effects of these disulfidptosis-related DEGs on EMs by enhancing cell migration, adhesion, and survival in EMs. In contrast to the genes mentioned above, PDLIM1 is found to be antagonistically downregulated in the ectopic endometrium. PDLIM1 is a tumour-suppressing gene that effectively blocks the Hippo-Yes-associated protein (YAP) signalling pathway [72]. But the overexpressed YAP signal can stimulate the proliferation and inhibit apoptosis of endometrial stromal cells in EMs [73]. This discovery provides evidence that decreased expression of PDLIM1 could lead to EMs by promoting cell proliferation in the ectopic lesions. Interestingly, it has also been revealed that PDLIM1 can promote the separation of ACTN4 from F-actin, consequently impeding F-actin overgrowth and suppressing cell migration [74]. Based on the aforementioned analysis, we can speculate that the synergistic or antagonistic interplays of these disulfidptosis-related DEGs are collectively involved in EMs, thus providing valuable insight for further research on disulfidptosis in EMs.

Additionally, previous studies indicate that the dysfunction of immune inflammation plays a crucial role in EMs [75]. Thus, we thoroughly depicted the immune infiltration landscape in both eutopic and ectopic endometrial tissue of EMs and explored the correlation of disulfidptosis-related DEGs with immunocytes of EMs. The abundance of multiple immune cells was abnormally altered in both situ and ectopic tissues, including macrophages, mast cells, NK cells, dendritic cells, neutrophils, plasma cells, and T cells. For example, an upregulation of M2 macrophages in the ectopic endometrial tissue was observed in our study. In general, macrophages serve as key effector cells in innate immunity and can be categorized into proinflammatory M1 phenotype and anti-inflammatory M2 phenotype [55]. Prior research has found highly expressed M2 macrophages in the ectopic lesions of EMS, suggesting that the presence of an anti-inflammatory milieu may be permissible for lesion formation and persistence [75]. In addition, we found activated mast cells were upregulated in both eutopic and ectopic endometrial tissues. Consistently, mast cell activation has been verified to secrete interleukin 1 (IL-1), TNF-α, and stem cell factor, which enhances the inflammatory response, fibrosis, and angiogenesis, accelerating endometrial cell migration and ectopic lesions formation [76, 77]. In addition, we also uncovered that the activated NK cells and dendritic cells were decreased in the ectopic endometrium of EMs. And it has been confirmed that dendritic cells inhibit neovascularization and progression in EMs [78], and the suppression of NK cells can reduce their cytotoxic activity and the elimination of ectopic endometrial cells [79]. Taken together, all of these findings support our results and highlight that the altered concentration of immune cells is a pivotal factor in the initiation and advancement of EMs.

We next explored the association of the disulfidptosis-related DEGs with immunocytes. Our data revealed that monocytes were positively connected to INF2 in the eutopic endometria tissues and SLC3A2 in the ectopic endometria tissues. INF2, a formin protein involved in both the polymerization and depolymerization of actin, is crucial for cell migration and adhesion [80, 81]. SLC3A2 has been domonstrated to enhance integrin-mediated cell migration and safeguard against apoptosis by activating Akt and Rac GTPase [82]. And cell migration, mediated by the actin cytoskeleton, is the prerequisite for immune cells to reach a specific site to perform immune function [83]. Notably, the process of monocyte migration and aggregation during inflammation is also partly facilitated by integrin [84, 85]. Interestingly, actin polymerization is a key factor in forming disulfide, which results in disulfidptosis [6]. Therefore, it may be hypothesized that the disulfidptosis-related DEGs in EMs may lead to an augmentation or reduction of pertinent immune cells in the local endometrial tissue through modulating the actin cytoskeleton and cell migration. However, there is still a scarcity of studies that explore the potential correlation between disulfidptosis and immune cells. Our research findings may offer a novel perspective for the specific biological mechanism of disulfidptosis in the immune landscape of EMs.

Due to the reliance on invasive procedures like laparoscopy and histopathology [86], the conventional diagnostic strategy of EMs inevitably causes delays in preemptive measures, which severely impacts the physical and mental health of women. Hence, exploring noninvasive and highly specific biomarkers holds immense importance for the prompt identification and management of EMs. Combining bioinformatics with machine learning provides a creative strategy, significantly improving diagnostic model precision. Given the particular involvement of disulfidptosis in EMs, we investigated prospective diagnostic biomarkers for EMs based on disulfidptosis-related DEGs in EMs. Through BLR, LASSO, SVM-RFE, and XGBoost, seven signature genes (ACTB, GYS1, IQGAP1, MYH10, NUBPL, SLC7A11, and TLN1) in eutopic endometria tissues and five signature genes (CAPZB, CD2AP, MYH10, OXSM, and PDLIM1) in ectopic endometria tissues displayed prominent diagnostic value. Subsequent independent validation sets further confirmed the validity and precision of IQGAP1 and SLC7A11 in eutopic endometria tissues, as well as MYH10, PDLIM1, and CD2AP in ectopic endometria tissues. IQGAP1 is a cytoplasmic scaffold protein with a key role in cell attachment, migration, and RAF (Raf serine/threonine kinases)/MEK (MAPK/ERK kinase)/ERK (extracellular signal-regulated kinase) signaling activation [87–89]. Coincidentally, RAF/MEK/ERK signaling activation strengthens endometrial cell proliferation and spread in EMs [90]. Regarding SLC7A11, studies have revealed that the up-regulation of SLC7A11 impeds the progression of EMs by triggering ferroptosis [91, 92]. Moreover, PDLIM1 suppresses the Hippo-YAP signaling pathway. Significantly, inhibition of the Hippo-YAP signaling pathway has been demonstrated to effectively hinder proliferation and enhance apoptosis of endometrial stromal cells [72, 73]. As for MYH10, it participates in epithelial-mesenchymal transition (EMT) [93]. EMT is considered a necessary condition for the onset of endometriotic foci, as EMT is characterized by the loss of polarity and intercelluar connections in epithelial cells, leading to the acquisition of migratory and invasive traits associated with mesenchymal cells [94, 95]. CD2AP is a crucial adhesion-associated adapter protein, and extensively expresses in the endometrium and endocervix [96]. It has been demenstrated to be involved in connecting membrane proteins with the actin cytoskeleton, thereby driving cell migration and adhesion, which is closely associated with the the ectopic lesion formation [97]. Overall, the above studies provide substantial foundation for identification of these genes as diagnostic genes for EMs. Notably, this is the first time we have underscored the predict value of IQGAP1, SLC7A11, MYH10, CD2AP, and PDLIM1 in the involvement of disulfidptosis in EMs. Of course, it has to be admitted that the diagnostic efficacy of the genes in our study was only verified in 2 independent validation sets (GSE141549 and GSE58178), and it is necessary to follow up the GEO database in the future to examine their diagnostic performance in more eligible independent validation sets. Moreover, it is necessary to examine the expression of diagnostic genes in prospective cohorts to provide sufficient evidence to promote them as markers in the clinic. Collectively, our research may offer novel inspiration for finding more accurate and reliable biomarkers for EMs diagnosis and treatment.

Based on the three disulfidptosis-related diagnostic biomarkers in the ectopic endometrial tissues of EMs validated by the independent validation dataset, 12 compounds potentially targeting these disulfidptosis-related signature genes were screened from the CTD, Drugbank, and DGIdb databases, including cisplatin, cyclosporine, estradiol, ethinyl estradiol, indomethacin, panobinostat, tretinoin, valproic acid, acetaminophen, doxorubicin, phenobarbital, and prednisone. Remarkably, tretinoin, a metabolite of vitamin A, is necessary for activating various immunocytes and serves as a vital regulator of immune inflammation [98]. Existing researches have verified the suppressive impacts of tretinoin on EMs in both cellular and animal experiments [99–101]. Specifically, tretinoin treatment could effectively suppress the onset and expansion of endometriotic lesions in EMs mice by stimulating pro-inflammatory macrophage differentiation, which exerts enhanced phagocytosis to eliminate ectopic endometrial tissue [102]. Furthermore, Hatice Ozer et al. demonstrated that tretinoin limited angiogenesis in EMs by inhibiting the production of VEGF, hence effectively shrinking the ectopic lesions [46]. Conversely, containment of tretinoin production could facilitate the appearance of an “endometriosis phenotype” that allows celluar implanation and proliferation at abnormal locations [103]. These studies support our findings and offer compelling justification for further investigation into the therapeutic potential of tretinoin in EMs. Thus, we further validated the theraputic feasibility and efficiency of tretinoin in the EMs murine model. And results depicted a considerable reduction in lesion volume, and the levels of CD31, VEGF, and inflammatory factors (IL-1β, TNF-α) in the tretinoin group. Furthermore, a significantly downregulated expression level of CD2AP and MYH10, as well as an upregulated expression level of PDLIM1, were observed in the tretinoin group. Given the major impact of disulfidptosis on EMs, we confidently propose that adopting tretinoin as a pharmaceutical selection based on its modulation on disulfidptosis may offer a novel therapeutic strategy for EMs. Taken together, our finding contributes to the novel treatment options for EMs, and broadens the insights into the original pharmacological effects of tretinoin, which also facilitates to amplify its clinical applications. Of course, large-scale clinical trials should be conducted for ascertaining the safe dosage and treatment course of tretinoin for EMs therapy.

## Conclusion

To summarize, our study is the first to probe the involvement of disulfidptosis in the pathomechanism of EMs and reveal the association between disulfidptosis and the immunological dysfunction present in both eutopic and ectopic endometrial tissues of EMs, utilizing in-depth bioinformatics analysis. Most importantly, this work constructed diagnosis models with prominent diagnostic efficacy in both eutopic and ectopic endometrial tissues by using multiple machine learning algorithms (BLR, LASSO, SVM-RFE, and XGBoost), the performances of which were determined in the testing sets. And seven eutopic signature genes and five ectopic signature genes were screened based on the diagnosis models mentioned above. This new diagnostic strategy that integrates multiple disulfidptosis-related biomarkers provides new insights on the timely and accurate diagnosis of EMs in a clinical setting. In addition, potential compounds targeting the signature genes were screened, and the pharmacological effect of tretinoin on lesion volume, inflammatory factors (IL-1β, TNF-α), and the expression of CD31, VEGF, and the signature genes were further verified in the ectopic lesion in the EMs murine model. Nevertheless, these findings are based on transcriptomic information from publicly available databases, so confounding factors such as race, sample size, etc. may inevitably interfere with the objectivity of the results. In addition, even though the diagnostic genes we screened were validated in independent validation sets and model animals, the large-scale clinical trails are needed to establish these signature genes as clinically universal diagnostic indicators.

### Supplementary Information

Supplementary Material 1: Supplement table 1. Immune localization of EMs signature genes.

Supplementary Material 2: Supplement table 2. Tests for normality distribution and homogeneity of variance.

Supplementary Material 3: Supplement figure 1. Correlation plot of eutopic disulfidptosis-related DEGs (A) and ectopic disulfidptosis-related DEGs (B) remaining after filtering out those with high correlation.

Supplementary Material 4: Supplement figure 2. ROC curve for BLR, LASSO, SVM-RFE, and XGBoost algorithms in relation to eutopic (A) and ectopic (B) tissues.

Supplementary Material 5: Supplement figure 3. The ROC curves of signature genes in eutopic and ectopic endometrial tissues, respectively. (A-G) The ROC of eutopic signature genes. (H-L) The ROC of ectopic signature genes.

Supplementary Material 6: Supplement figure 4. Candidate compound screening. The association network of signature genes with medicine. Core nodes: signature genes. Peripheral nodes: medicine. The Approved drug node color is yellow-red, the Approved and Investigational drug node color is purple-pink, the Approved, Investigational and Nutraceutical drug node color is purple-red, the Approved, Investigational and Vet approved drug node color is yellow-pink, the Approved and Vet approved drug node color is pink.

## Data Availability

The original contributions presented in the study are included in the article/supplementary material, further inquiries can be directed to the corresponding authors.

## Abbreviations

EMs: Endometriosis
DRGs: Disulfidptosis-related genes
PPI network: Protein-protein interaction network
VEGF: Vascular endothelial growth factor
TNF-α: Tumor necrosis factor alpha
IL-1β: Interleukin-1beta
SLC7A11: Solute carrier family 7 member 11
NADPH: Nicotinamide adenine dinucleotide phosphate
GEO: Gene Expression Omnibus
WGCNA: Weighted gene co-expression network analysis
DEGs: Differentially expressed genes
GO: Gene Ontology
GO-CC: Gene Ontology-Cellular Component
GO-BP: Gene Ontology-Biological Process
GO-MF: Gene Ontology-Molecular Function
KEGG: Kyoto Encyclopedia of Genes and Genomes
FDA: Food and Drug Administration
TOM: Topological Overlap Measure
RMA: Multi-array analysis
HLA: Human leukocyte antigen
BLR: Binary logistic regression
SVM-RFE: Support vector machine-recursive feature elimination
LASSO: Least absolute shrinkage and selection operator
XGBoost: Extreme gradient boosting
ROC: Receiver operating characteristic
AUC: Area under curve
PPV: Positive predictive value
NPV: Negative predictive value
CTD: Comparative Toxicogenomics Database
DGIdb: Drug-gene interaction database
qRT-PCR: Quantitative real-time polymerase chain reaction
DAPI: 4',6-diamidino-2-phenylindole
ANOVA: Analysis of variance
PI3K: Phosphatidylinositol 3-kinase
AKT: Protein kinase B
TLRs: Toll-like receptors
TNF: Tumor necrosis factor
ICAM-1: Intercellular adhesion molecule-1
VCAM-1: Vascular cellular adhesion molecule-1
pAKT: Phosphorylated AKT
MMP-2: Matrix metalloproteinase-2
ERK: Extracellular signal-regulated kinase
mTORC2: Mechanistic target of rapamycin complex 2
Hippo-YAP: Hippo-Yes-associated protein
IL-1: Interleukin 1
IL-8: Interleukin 8
EMT: Epithelial-mesenchymal transition
Raf: Raf serine/threonine kinases
MEK: MAPK/ERK kinase
