# Supplementary material for: Unraveling pathogenesis, biomarkers and potential therapeutic agents for endometriosis associated with disulfidptosis based on bioinformatics analysis, machine learning and experiment validation
Source: J Biol Eng. 2024 Jul 26;18:42. doi: 10.1186/s13036-024-00437-0 (PMC11282767; doi:10.1186/s13036-024-00437-0)
Supplement: Supplementary file 1 — Supplementary Material 1: Supplement table 1. Immune localization of EMs signature genes. [file 13036_2024_437_MOESM1_ESM.doc]

Supplement table 1. Immune localization of EMs signature genes

| Gene | Description  (referring to the Genecards database) | Expression in uterine tissue (referring to the Human Protein Atlas database) | Expression in immocytes (referring to the bioGPS database) | Subcellular summary (referring to the Human Protein Atlas database) | Function (referring to the bioGPS, Genecards, Alliance of Genome Resources and Uniprot database) | The localization of differentially expressed genes |
| --- | --- | --- | --- | --- | --- | --- |
| ACTB | Actin Beta | YES | NK cell, B cell, Dentritic cell, CD4+T cell, CD8+T cell, Monocyte | - | ACTB is involved in cell motility, structure, and integrity (PMID:25788699, PMID:6842590, PMID:[29581253](https://pubmed.ncbi.nlm.nih.gov/29581253/)). | Eutopic endometrium |
| ACTN4 | Actinin Alpha 4 | YES | NK cell, Dentritic cell, Monocyte | Actin filaments and Cytosol | ACTN4 potentiates transcriptional activation by MEF2 transcription factors, estrogen receptor α, and vitamin D3 receptor (PMID:22351778). It also functions as a transcriptional coactivator, stimulating transcription mediated by the nuclear hormone receptors PPARG and RARA (PMID:22351778). | Ectopic endometrium, Eutopic endometrium |
| CAPZB | Capping Actin Protein Of Muscle Z-Line Subunit Beta | YES | B cell, Dentritic cell, CD4+T cell, CD8+T cell, Monocyte | Nucleoplasm, Cytosol and Vesicles | CAPZB functions to cap actin filaments at barbed (+) ends, thus controlling the rate of G-actin polymerization to F-actin and the corresponding filament length (PMID:7822423). | Ectopic endometrium, Eutopic endometrium |
| CD2AP | CD2 Associated Protein | YES | NK cell, B cell, Dentritic cell, CD4+T cell, CD8+T cell | Plasma membrane and Centriolar satellite | CD2AP acts as an adapter protein between membrane proteins and the actin cytoskeleton and is also required for cytokinesis (PMID:10339567, PMID:15800069). | Ectopic endometrium, Eutopic endometrium |
| DSTN | Destrin, Actin Depolymerizing Factor | YES | NK cell | Plasma membrane | Destrin regulates actin in the cytoskeleton and can bind actin. The binding of actin by destrin and cofilin is negatively regulated by phosphorylation. Furthermore, destrin can also sever actin filaments (PMID:8674111). | Ectopic endometrium, Eutopic endometrium |
| FLNA | Filamin A | YES | NK cell, B cell, Dentritic cell, CD4+T cell, CD8+T cell, Monocyte | Plasma membrane, Actin filaments and Cytosol | FLNA mediates the positioning of the actin cytoskeleton (PMID: 22121117).  During the axon guidance process, FLNA is required for growth cone collapse induced by SEMA3A-mediated stimulation of neurons (PubMed:25358863). | Ectopic endometrium, Eutopic endometrium |
| FLNB | Filamin B | YES | Dentritic cel | Plasma membrane, Golgi apparatus, Actin filaments and Cytosol | FLNB regulates intracellular communication and signalling by cross-linking the protein actin to allow direct communication between the cell membrane and cytoskeletal network and to control and guide proper skeletal development (PMID:17510210). | Ectopic endometrium |
| GYS1 | Glycogen Synthase 1 | YES | NK cell, B cell, Dentritic cell, CD4+T cell, CD8+T cell, Monocyte | Microtubules and Cytosol | GYS1 is vital for glycogen synthesis in heart and muscle, giving a reserve of critical energy during bursts of activity, sustained muscle work and hypoxia (PMID:19699667). | Eutopic endometrium |
| INF2 | Inverted Formin 2 | YES | B cell, CD4+T cell | Endoplasmic reticulum and Nuclear bodies | INF2 plays a role in mitochondrial fission and dorsal stress fiber formation (PMID:23349293). It can accelerate actin nucleation and elongation by interacting with barbed ends (fast-growing ends) of actin filaments, but it can also accelerate the disassembly of actin through encircling and severing filaments (PMID:24412206). | Eutopic endometrium |
| IQGAP1 | IQ Motif Containing GTPase Activating Protein 1 | YES | NK cell, B cell, Dentritic cell, CD4+T cell, CD8+T cell, Monocyte | Plasma membrane and Cell junctions | IQGAP interacts with components of the cytoskeleton such as the formin Dia1 (mDia1), with cell adhesion molecules (CAMs), and with several signaling molecules to regulate cell morphology and motility (PMID:17972901, PMID:17620407). It may play a possible role in cell cycle regulation by contributing to cell cycle progression after DNA replication arrest (PMID:20883816). | Eutopic endometrium |
| LRPPRC | Leucine Rich Pentatricopeptide Repeat Containing | YES | NK cell, B cell, Dentritic cell, CD4+T cell, CD8+T cell, Monocyte | Mitochondria | LRPPRC is thought to play a role in regulating the interaction of the cytoskeleton with a variety of cellular processes (PMID:11827465).  It may play a role in RNA metabolism in both nuclei and mitochondria (PMID:19262567, PMID:28325843) and can also bind to exportin XPO1/CRM1 to engage the nuclear pore and traffic the bound mRNAs to the cytoplasm (PMID:28325843). | Ectopic endometrium, Eutopic endometrium |
| MYH10 | Myosin Heavy Chain 10 | YES | - | Actin filaments, Mitochondria and Cytosol | MYH10 appears to play a role in cytokinesis, cell shape, and specialized functions such as secretion and capping. During cell spreading, it plays an important role in cytoskeleton reorganization, focal contacts formation (in the central part but not the margins of spreading cells), and lamellipodial extension (PMID: 20052411, PMID: 20603131). | Ectopic endometrium, Eutopic endometrium |
| MYH9 | Myosin Heavy Chain 9 | YES | NK cell, B cell, Dentritic cell, CD4+T cell, CD8+T cell, Monocyte | Plasma membrane, Actin filaments, Nuclear bodies and Cytosol | MYH9 promotes cell motility in conjunction with S100A4 (PMID:16707441). During cell spreading, it plays an important role in cytoskeleton reorganization, focal contact formation (in the margins but not the central part of spreading cells), and lamellipodial retraction (PMID:20052411). | Ectopic endometrium, Eutopic endometrium |
| NCKAP1 | NCK Associated Protein 1 | YES | - | Cytosol | NCKAP1 contributes to small GTPase-binding activity. It is also involved in Rac protein signal transduction, positive regulation of Arp2/3 complex-mediated actin nucleation, and positive regulation of lamellipodium assembly (provided by the Alliance of Genome Resources, April 2022). | Eutopic endometrium |
| NDUFS1 | NADH:Ubiquinone Oxidoreductase Core Subunit S1 | YES | NK cell, B cell, Dentritic cell, CD4+T cell, CD8+T cell, Monocyte | Mitochondria | NDUFS1 is the core subunit of the mitochondrial membrane respiratory chain NADH dehydrogenase (Complex I), which catalyzes electron transfer from NADH through the respiratory chain using ubiquinone as an electron acceptor (PMID:30879903, PMID:31557978). It is essential for catalysing the entry and efficient transfer of electrons within complex I and plays a key role in the assembly and stability of complex I (PMID:31557978, PMID:30879903, PMID:31557978). | Ectopic endometrium, Eutopic endometrium |
| NUBPL | NUBP Iron-Sulfur Cluster Assembly Factor, Mitochondrial | YES | NK cell, B cell, Dentritic cell, CD4+T cell, Monocyte | Mitochondria | The NUBPL is required for the assembly of the mitochondrial membrane respiratory chain NADH dehydrogenase (Complex I), the first oligomeric enzymatic complex of the mitochondrial respiratory chain located in the inner mitochondrial membrane (PMID: 19752196). Its role in assembly is the delivery of one or more iron-sulfur (Fe-S) clusters to complex I subunits in anaerobic conditions in vitro (PMID:19752196). | Eutopic endometrium |
| OXSM | 3-Oxoacyl-ACP Synthase, Mitochondrial | YES | NK cell, B cell, Dentritic cell, CD4+T cell, CD8+T cell | Mitochondria and Cytosol | OXSM may play a role in the biosynthesis of lipoic acid as well as longer chain fatty acids required for optimal mitochondrial function (PMID: 15668256). | Ectopic endometrium, Eutopic endometrium |
| PDLIM1 | PDZ And LIM Domain 1 | YES | NK cell, B cell, Dentritic cell | Actin filaments, Plasma membrane and Cell junctions | PDLIM1 acts as an adapter that brings other proteins (like kinases) to the cytoskeleton (PMID:10861853). | Ectopic endometrium, Eutopic endometrium |
| RPN1 | Ribophorin I | YES | NK cell, B cell, Dentritic cell, CD4+T cell, Monocyte | Endoplasmic reticulum and Cytosol | RPN1 is subunit of the oligosaccharyl transferase (OST) complex that catalyzes the initial transfer of a defined glycan (Glc3Man9GlcNAc2 in eukaryotes) from the lipid carrier dolichol-pyrophosphate to an asparagine residue within an Asn-X-Ser/Thr consensus motif in nascent polypeptide chains, the first step in protein N-glycosylation (PMID:31831667). | Ectopic endometrium |
| SLC3A2 | Solute Carrier Family 3 Member 2 | YES | NK cell, B cell, Dentritic cell, Monocyte | Nucleoplasm and Plasma membrane | SLC3A2 facilitates the biogenesis and trafficking of functional transporter heterodimers to the plasma membrane and mediates the uptake of dibasic amino acids (PMID:9829974, PMID:10903140).  It also functions as an antiporter by mediating the exchange of extracellular anionic L-cystine and intracellular L-glutamate across the cellular plasma membrane (PMID:34880232) and modulates integrin-related signaling and is essential for integrin-dependent cell spreading, migration and tumor progression (PMID:15625115, PMID:11121428). | Ectopic endometrium, Eutopic endometrium |
| SLC7A11 | Solute Carrier Family 7 Member 11 | YES | NK cell, B cell, Dentritic cell, CD4+T cell, Monocyte | Vesicles | SLC7A11 functions as an antiporter by mediating the exchange of extracellular anionic L-cystine and intracellular L-glutamate across the cellular plasma membrane (PMID:15151999, PMID:34880232).  It mediates the import of L-kynurenine, leading to the anti-ferroptotic signaling propagation required to maintain L-cystine and glutathione homeostasis (PMID:35245456) and mediates N-acetyl-L-cysteine uptake into the placenta, subsequently down-regulating pathways associated with oxidative stress, inflammation, and apoptosis (PMID:34120018). | Ectopic endometrium, Eutopic endometrium |
| TLN1 | Talin 1 | YES | NK cell, B cell, Dentritic cell, Monocyte | Focal adhesion sites, Cytosol, Plasma membrane and Centriolar satellite | The primary function of TLN1 involves the linkage of integrins to the actin cytoskeleton and the energy-dependent activation of integrins (PMID:19416068, PMID:14754902). | Ectopic endometrium, Eutopic endometrium |
