# Supplementary material for: Unraveling pathogenesis, biomarkers and potential therapeutic agents for endometriosis associated with disulfidptosis based on bioinformatics analysis, machine learning and experiment validation
Source: J Biol Eng. 2024 Jul 26;18:42. doi: 10.1186/s13036-024-00437-0 (PMC11282767; doi:10.1186/s13036-024-00437-0)
Supplement: Supplementary file 2 — Supplementary Material 2: Supplement table 2. Tests for normality distribution and homogeneity of variance. [file 13036_2024_437_MOESM2_ESM.doc]

Supplement table 2. Tests for normality distribution and homogeneity of variance

1. The normality of the data was tested using the Shapiro-Wilk test

| Figure 12A | Group: EMT | Group: EMT+Tre |  |
| --- | --- | --- | --- |
| W | 0.9826 | 0.9643 |  |
| P valve | 0.7470 | 0.6369 |  |
| Passed normality test (alpha=0.05)? | Yes | Yes |  |
| P valve summary | ns | ns |  |
| Figure 12B (relative IL-1β mRNA expression) | Group: Control | Group: EMT | Group: EMT+Tre |
| W | 0.9256 | 0.9041 | 0.9195 |
| P valve | 0.5463 | 0.3985 | 0.5015 |
| Passed normality test (alpha=0.05)? | Yes | Yes | Yes |
| P valve summary | ns | ns | ns |
| Figure 12B (relative TNF-α mRNA expression) | Group: Control | Group: EMT | Group: EMT+Tre |
| W | 0.9460 | 0.9440 | 0.9217 |
| P valve | 0.7078 | 0.6920 | 0.5177 |
| Passed normality test (alpha=0.05)? | Yes | Yes | Yes |
| P valve summary | ns | ns | ns |
| Figure 12D (CD31) | Group: Control | Group: EMT | Group: EMT+Tre |
| W | 0.8530 | 0.9980 | 0.9395 |
| P valve | 0.2485 | 0.9137 | 0.5255 |
| Passed normality test (alpha=0.05)? | Yes | Yes | Yes |
| P valve summary | ns | ns | ns |
| Figure 12E (VEGF) | Group: Control | Group: EMT | Group: EMT+Tre |
| W | 0.8126 | 0.9552 | 0.9365 |
| P valve | 0.1449 | 0.5926 | 0.5136 |
| Passed normality test (alpha=0.05)? | Yes | Yes | Yes |
| P valve summary | ns | ns | ns |
| Figure 12F (MYH10) | Group: Control | Group: EMT | Group: EMT+Tre |
| W | 0.8852 | 0.9091 | 0.7765 |
| P valve | 0.3399 | 0.4151 | 0.0595 |
| Passed normality test (alpha=0.05)? | Yes | Yes | Yes |
| P valve summary | ns | ns | ns |
| Figure 12G (CD2AP) | Group: Control | Group: EMT | Group: EMT+Tre |
| W | 0.9672 | 0.9910 | 0.9943 |
| P valve | 0.6523 | 0.8187 | 0.8555 |
| Passed normality test (alpha=0.05)? | Yes | Yes | Yes |
| P valve summary | ns | ns | ns |
| Figure 12H (PDLIM1) | Group: Control | Group: EMT | Group: EMT+Tre |
| W | 0.9282 | 0.9981 | 0.9881 |
| P valve | 0.4818 | 0.9168 | 0.7916 |
| Passed normality test (alpha=0.05)? | Yes | Yes | Yes |
| P valve summary | ns | ns | ns |

1. The variance homogeneity of the data was tested using the Brown-forsythe test

| Figure 12B (relative IL-1β mRNA expression) |  |
| --- | --- |
| F | 0.8309 |
| P valve | 0.4547 |
| P valve summary | ns |
| Are SDs significantly different (P<0.05)? | No |
| Figure 12B (relative TNF-α mRNA expression) |  |
| F | 2.576 |
| P valve | 0.1092 |
| P valve summary | ns |
| Are SDs significantly different (P<0.05)? | No |
| Figure 12D (CD31) |  |
| F | 0.7696 |
| P valve | 0.5041 |
| P valve summary | ns |
| Are SDs significantly different (P<0.05)? | No |
| Figure 12E (VEGF) |  |
| F | 0.4022 |
| P valve | 0.6856 |
| P valve summary | ns |
| Are SDs significantly different (P<0.05)? | No |
| Figure 12F (MYH10) |  |
| F | 0.6999 |
| P valve | 0.5331 |
| P valve summary | ns |
| Are SDs significantly different (P<0.05)? | No |
| Figure 12G (CD2AP) |  |
| F | 0.1302 |
| P valve | 0.8803 |
| P valve summary | ns |
| Are SDs significantly different (P<0.05)? | No |
| Figure 12H (PDLIM1) |  |
| F | 0.1709 |
| P valve | 0.8469 |
| P valve summary | ns |
| Are SDs significantly different (P<0.05)? | No |
