# Supplementary figures and images for: Unraveling pathogenesis, biomarkers and potential therapeutic agents for endometriosis associated with disulfidptosis based on bioinformatics analysis, machine learning and experiment validation
Source: J Biol Eng. 2024 Jul 26;18:42. doi: 10.1186/s13036-024-00437-0 (PMC11282767; doi:10.1186/s13036-024-00437-0)

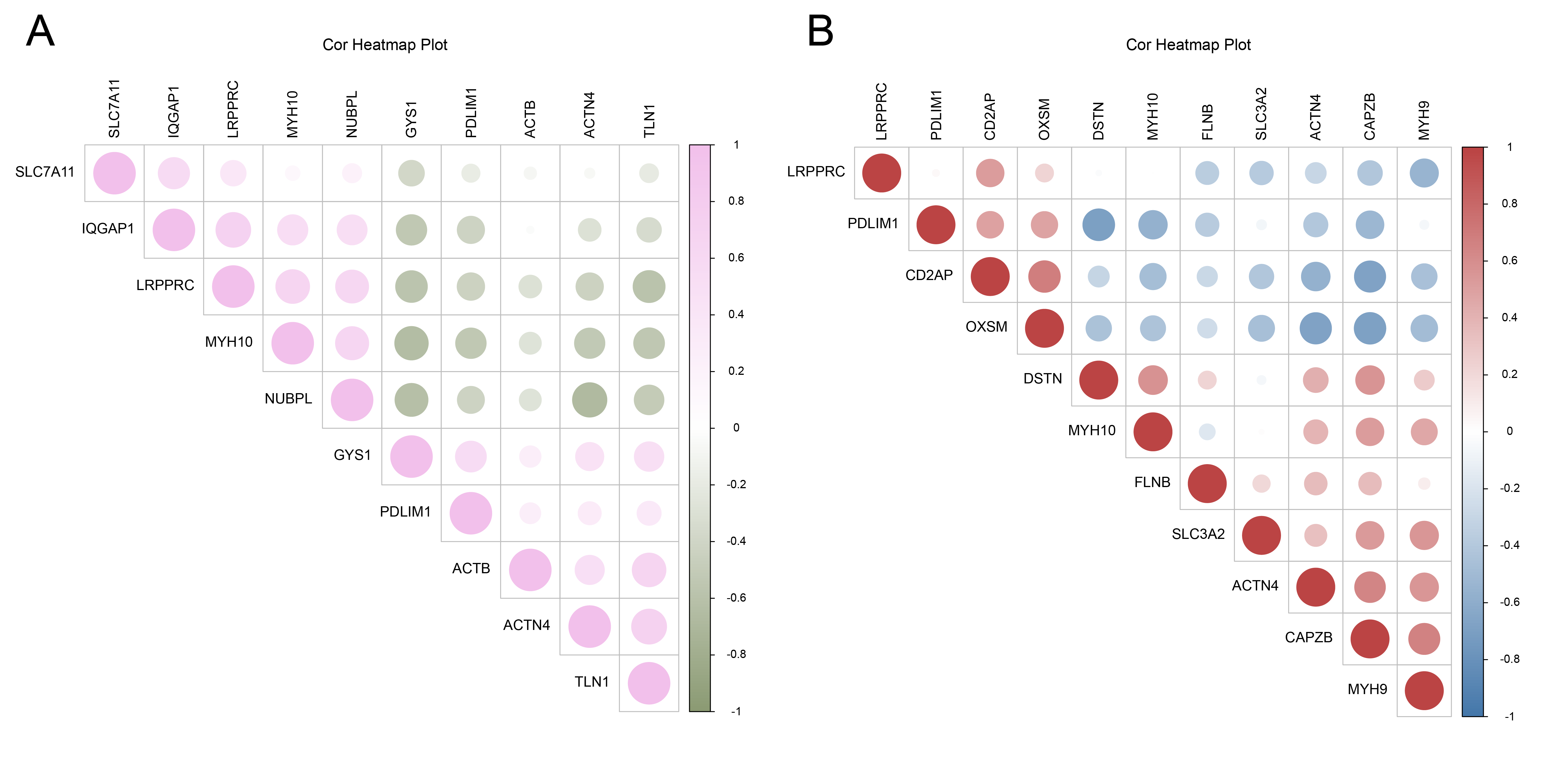

Supplement: Supplementary file 3 — Supplementary Material 3: Supplement figure 1. Correlation plot of eutopic disulfidptosis-related DEGs (A) and ectopic disulfidptosis-related DEGs (B) remaining after filtering out those with high correlation. [file 13036_2024_437_MOESM3_ESM.tif]

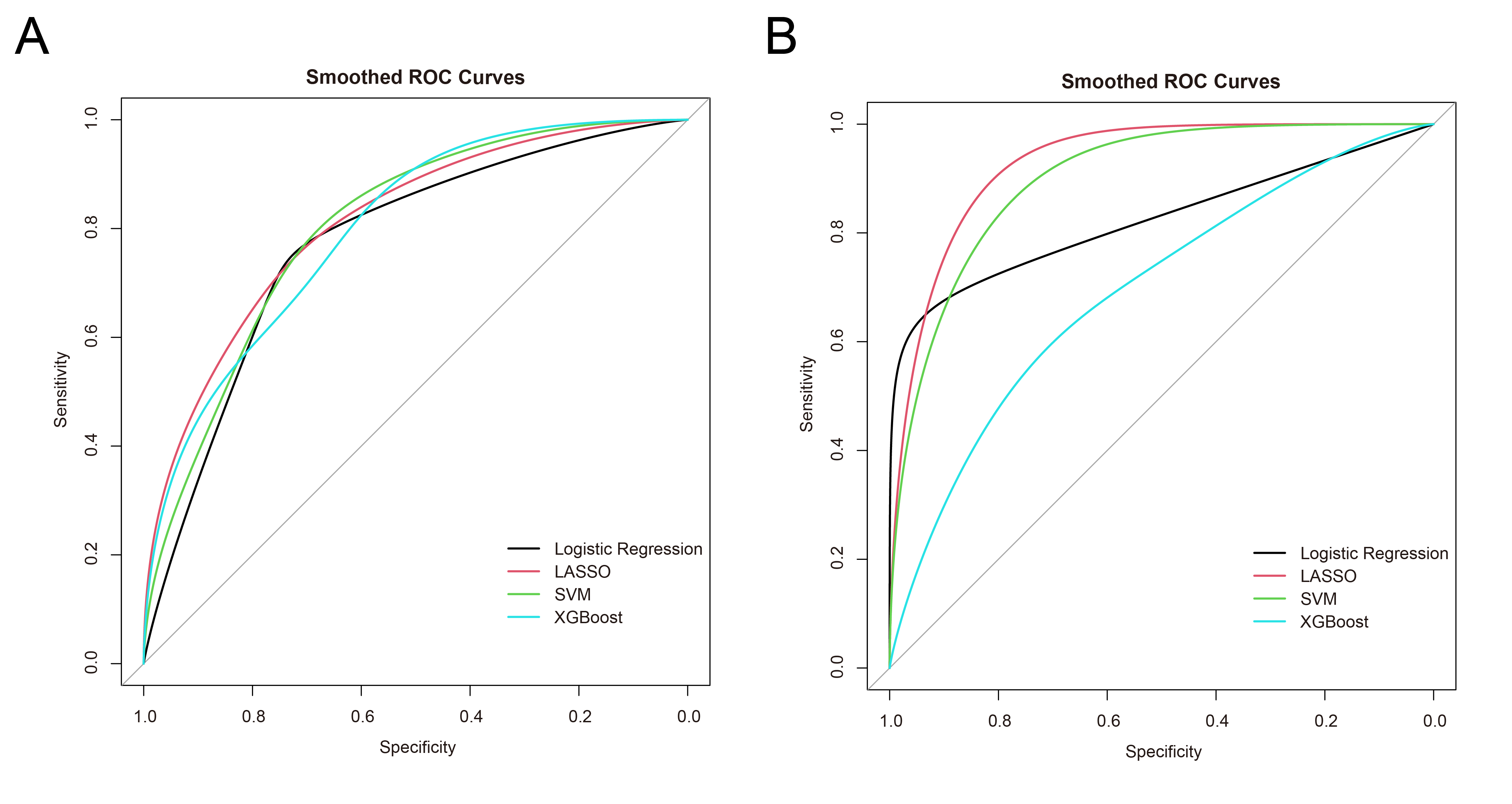

Supplement: Supplementary file 4 — Supplementary Material 4: Supplement figure 2. ROC curve for BLR, LASSO, SVM-RFE, and XGBoost algorithms in relation to eutopic (A) and ectopic (B) tissues. [file 13036_2024_437_MOESM4_ESM.tif]

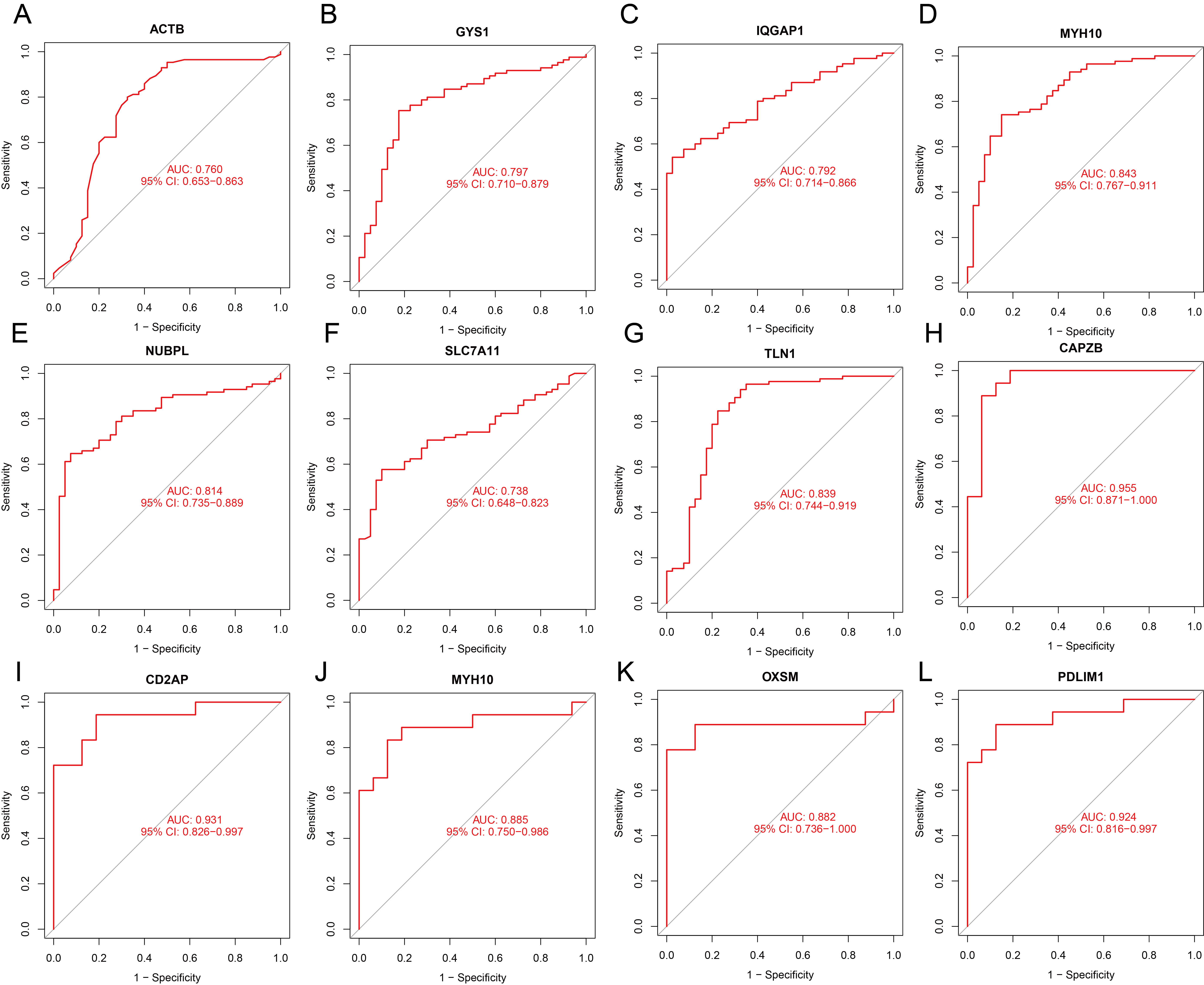

Supplement: Supplementary file 5 — Supplementary Material 5: Supplement figure 3. The ROC curves of signature genes in eutopic and ectopic endometrial tissues, respectively. (A-G) The ROC of eutopic signature genes. (H-L) The ROC of ectopic signature genes. [file 13036_2024_437_MOESM5_ESM.tif]

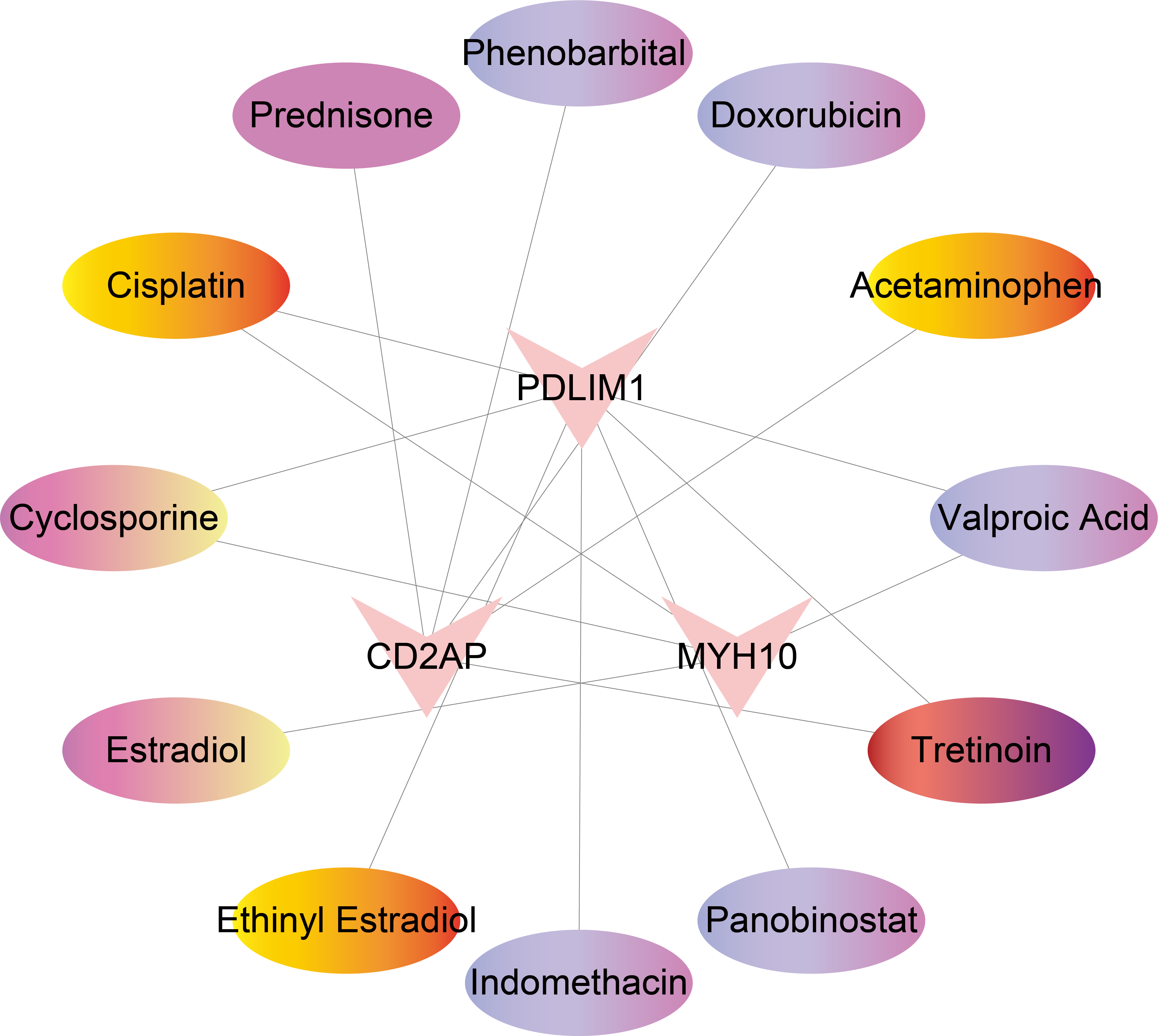

Supplement: Supplementary file 6 — Supplementary Material 6: Supplement figure 4. Candidate compound screening. The association network of signature genes with medicine. Core nodes: signature genes. Peripheral nodes: medicine. The Approved drug node color is yellow-red, the Approved and Investigational drug node color is purple-pink, the Approved, Investigational and Nutraceutical drug node color is purple-red, the Approved, Investigational and Vet approved drug node color is yellow-pink, the Approved and Vet approved drug node color is pink. [file 13036_2024_437_MOESM6_ESM.tif]
